# Art by firelight? Using experimental and digital techniques to explore Magdalenian engraved plaquette use at Montastruc (France)

**DOI:** 10.1371/journal.pone.0266146

**Published:** 2022-04-20

**Authors:** Andy Needham, Izzy Wisher, Andrew Langley, Matthew Amy, Aimée Little

**Affiliations:** 1 YEAR Centre, Department of Archaeology, University of York, York, United Kingdom; 2 Department of Archaeology, Durham University, Durham, United Kingdom; Griffith University, AUSTRALIA

## Abstract

Palaeolithic stone plaquettes are a type of mobiliary art featuring engravings and recovered primarily from Magdalenian sites, where they can number from single finds to several thousand examples. Where context is available, they demonstrate complex traces of use, including surface refreshing, heating, and fragmentation. However, for plaquettes with limited or no archaeological context, research tends to gravitate toward their engraved surfaces. This paper focuses on 50 limestone plaquettes excavated by Peccadeau de l’Isle from Montastruc, a Magdalenian rockshelter site in southern France with limited archaeological context; a feature common to many art bearing sites excavated across the 19th and early 20th Centuries. Plaquette use at Montastruc was explored via a programme of microscopy, 3D modelling, colour enhancement using DStretch©, virtual reality (VR) modelling, and experimental archaeology, the latter focusing on limestone heating related to different functional and non-functional uses. While the limited archaeological context available ensures the results remain only indicative, the data generated suggests plaquettes from Montastruc were likely positioned in proximity to hearths during low ambient light conditions. The interaction of engraved stone and roving fire light made engraved forms appear dynamic and alive, suggesting this may have been important in their use. Human neurology is particularly attuned to interpreting shifting light and shadow as movement and identifying visually familiar forms in such varying light conditions through mechanisms such as pareidolic experience. This interpretation encourages a consideration of the possible conceptual connections between art made and experienced in similar circumstances, such as parietal art in dark cave environments. The toolset used to investigate the Montastruc assemblage may have application to other collections of plaquettes, particularly those with limited associated context.

## Introduction

Stone plaquettes—a type of portable art which can be defined in simple terms as having a tabular surface flat enough to support engraving—were a diverse artistic phenomenon in the Upper Palaeolithic. They feature a breadth of engraved or painted depictions, including: figurative or stylised animals [1–7]; humans and anthropomorphic forms, usually highly stylised [8–12]; abstract or geometric motifs [13]; and more rarely aspects of the environment or habitation areas, such as rivers [14] or built structures [15]. Plaquettes are found in greatest frequency in Western Europe, including Portugal to the southwest [16–18], Jersey [19] and Normandy [20] to the northwest, and with high concentrations in France, Spain and Germany [13, 21]. Plaquettes are only rarely reported from Central and Eastern Europe [22–24] and are absent from Britain, despite the presence of other types of Magdalenian parietal and portable art [21, 25–29].

Where archaeological context is available, plaquettes are implicated in a diverse range of activities. They may have been used in a variety of practical functions alongside undecorated examples in some instances, for example as pavement to stabilise surfaces at Enlène, Gönnersdorf, Roc-la-Tour I, Tito Bustillo, Las Caldas, and Urtiaga [8, 30–37]; as stone lamps [38–44]; and associated with fire or hearth structures at Ètiolles [45], Labastide [38, 46], La Marche [32], Mas d’Azil [32, 38], Limeuil [47, 48], and Enlène [38]. At a number of sites (see [30] for a comprehensive list) they may have been intentionally broken, such as at Las Caldas [34], Limeuil [48], Isturitz [38, 49], Cueva de Ekain [31, 50], Enlène [51] and Labastide [51]; and in some cases fragmented plaquettes were refreshed and engraved anew, such as at Foz do Medal Terrace [16, 17].

Yet where context is limited or absent, it is more difficult to assess their use or understand the relationship between plaquettes and other types of artefacts. Perhaps as a result, research efforts tend to focus on the engravings themselves. This is particularly true of sites excavated in the 19th and early 20th Centuries, where there is typically limited recording of spatial information, particularly with regard to which objects were found in a given layer or the relationship between certain artefacts and site features.

A continuing challenge facing Palaeolithic art studies is the development of approaches which can facilitate the deeper analysis of these archival objects that have limited contextual information. With the proliferation of digital and scientific techniques in recent decades, it may be possible to go further in the analysis of some archival artefacts. To explore potential solutions to this problem, the paper focuses on the high-resolution analysis of not only engravings, but also other features of plaquettes which may provide insight into material selection, artistic choices, use, or deposition. In this case, a combination of macroscopic and microscopic observation, experimental archaeology, 3D modelling, DStretch©, and virtual reality (VR) modelling are used. These tools are applied to the Peccadeau de l’Isle collection of limestone plaquettes from the Late Upper Palaeolithic site of Montastruc, curated in the British Museum. The plaquettes were excavated in the mid-19th century and have limited associated archaeological context. By way of interpretation, results suggest these plaquettes would have been placed in close proximity to hearth structures in low light levels, perhaps as a means of emphasising the relationships between engraved forms and natural features in the rock, with the dynamic light cast from a hearth bringing the depictions to life. This dynamic environment in which art was created and experienced ratchets with aspects of human neurology relating to the recognition of form and movement, including pareidolic experiences, making the production and use of plaquettes a visceral experience. No permits were required for the described study, which complied with all relevant regulations.

## Montastruc background

### Summary of the site, excavations, and artefacts

Montastruc is a Middle to Late Magdalenian rockshelter site located beneath a 29m high limestone cliff exposure, adjacent to the river Aveyron in the department of Tarn-et-Garonne, Southern France. Radiocarbon dating of a worked piece of antler and an antler spear tip yielded dates of 12070±180 BP (14587–13579 cal BP) and 13020±130 BP (15980–15220 cal BP). However, as the dating programme was undertaken in 1969, these results should be treated with caution [52]. The presence of a number of other sites found nearby including l’abri Plantade, l’abri Lafaye, l’abri Gandil and Courbet cave [21, 33, 53–59] suggests this area—and perhaps especially the rock shelters below the limestone cliffs—was important during the Magdalenian.

Montastruc was excavated by Peccadeau de l’Isle [54] in 1864 and 1866–1867. The artefacts recovered during these excavations are now stored in the British Museum (UK), while unmodified animal bones are stored in the Natural History Museum (UK). De l’Isle recognised 12 distinct levels and suggested that bone bearing deposits measured 6-7m deep across the excavated area [54]. Stratigraphy was composed of a succession of sands with ash and charcoal, and silts with red pebbles, the latter argued to be the result of flooding of the river Aveyron likely leading to phases of site abandonment [54]. The faunal assemblage recovered by de l’Isle included reindeer, deer, horse, ‘oxen’ (likely bison/aurochs), ‘goat’ (likely chamois/ibex), saïga, bear, wolf, fox and beaver, but also species grouped into the more general categories of ‘mammals’, ‘birds’ and ‘fish’ [54]. The stone tool assemblage was composed of 14,520 pieces and included bladelets (9,712), blades (1,228), burins (1,214), burin spalls (659), endscrapers (499), debitage (499), points (279) and combination tools (191) [60]. De l’Isle recovered organic portable art and stone plaquettes, perhaps most famous amongst them being the swimming reindeer made in ivory [21, 52] and an antler spearthrower with mammoth design [21, 39]. Decorated organic objects include double bevelled points (55), barbed points and harpoons (50), baguette demi ronde (9), perforated batons (8), personal ornaments (5), rondelles (5), spear throwers (3), contour découpé (1), as well as broken animal bones that were subsequently engraved to incorporate the irregular edges created by the break (4) [21, 60, 61]. An assemblage of 54 plaquettes was also recovered, primarily made on limestone and featuring naturalistic animal engravings.

Bernard Bétirac [53] excavated Montastruc between 1946–1947 and 1956–1957 [55]. The 1946–1947 excavation was published [53], but the 1956–1957 excavation was not. Artefacts from the excavations are curated in Le Musée d’Histoire Naturelle Victor Brun, Montaubon, and Musée Archéologie Nationale, Saint-Germain-en-Laye. Bétirac’s report gives a clearer sense of the stratigraphy and the relationship to material culture, providing a summary by layer (Table 1). The disparity between de l’Isle’s and Bétirac’s stratigraphy may be the result of truncation of the sequence by the former, likely due to clearance for the production of a railway embankment, followed by the preferential removal of sediment from the back of the rockshelter. Bétirac’s stratigraphic sequence suggests phases of periodic fluvial action in the rockshelter, rendering it periodically unsuitable for occupation, corroborating de l’Isle’s observations. This affects Montastruc at the base of the sequence, likely precluding earlier Magdalenian activity [53].

**Table 1:** 

Layer	Depth (metres)	Sediments	Artefacts	Fauna	Culture/ Age
VII	Not specified	No specifics noted	Sterile	Sterile	Sterile
VI	5.60-not specified	No specifics noted	Sterile	Sterile	Sterile
V	5.55–5.60	Entire layer composed of a thin layer of white ash	No specifics noted	No specifics noted	Mesolithic?
IV	5.00–5.55	Sandy texture, black in colour and free of scree. Evidence for fire cracked rock.	Rich in Late Magdalenian finds	Primarily reindeer and various fish. Horse, chamois, boar and red deer are found in less abundance. Targeting of young animals.	‘Magdalenian V and VI’
III	4.35–5.00	Sandy with inclusion of limestone blocks	Some bones and numerous lithics, possible workshop in this layer with a number of flakes found around a large stone	No specifics noted	No cultural attribution noted
II	4.20–4.35	Reddish in colour	Rich in lithics and bones	Reindeer and horse dominant, with ’large bovid’, red deer, ibex. Fox, marmot, hare, willow ptarmigan, black grouse and red billed chough	‘Magdalenian IV’
I	3.40–4.20	Sandy and laid down by fluvial action	May have been washed out due to high energy conditions	May have been washed out due to high energy conditions	‘Magdalenian III?’

Table summarising the stratigraphy and material culture recorded by Bétirac. Information derived from [53, 55].

Bétirac reported stone tools from the 1946–1947 excavation, numbering 3,467 utilised pieces, which included burins, endscrapers, combination tools, piercers and cores [53]. Bétirac [53] also recovered organic art and engraved stone plaquettes. This included a spearthrower with horse design [62, 63] and uniserial and biserial harpoons recovered from layers II and IV (see also [64] for an additional example from these excavations). No comprehensive catalogue of the stone plaquettes exist, but his summary [21, 53] suggests they are consistent with those recovered by de l’Isle; naturalistic and schematic animal designs engraved on supports of limestone likely derived from the site itself.

### Plaquettes from the Peccadeau de l’Isle collection, British Museum

Plaquettes found by de l’Isle number 54 examples, 50 of which were made on limestone, two from dark grey rounded river pebbles of unknown geology, and two of a metamorphosed laminar light grey stone of unknown geology. This includes a previously unreported limestone plaquette attributable to the Peccadeau de l’Isle collection identified by Jill Cook [60]. Detailed discussion of each plaquette has been published elsewhere [21, 60] and is summarised in Table 2. The limestone used in making plaquettes reflects careful and deliberate selection. The cliffs flanking the river Aveyron are part of a deep valley system cutting into a limestone plateau of Jurassic age. Weathered limestone likely derived from these cliffs was favoured [13], with blocks typically below 20cm in maximum dimension and carefully selected over other available materials nearby, such as waterworn pebbles [60].

**Table 2:** 

Museum no.	Raw material	Dimensions (L/W/D, cm)	Engraving	Heating
Palart.658	Limestone (pebble, tabular fragment)	10.8 x 9.5 x 1.8	Obverse: Bison (1) Reverse: Schematic lines	Obverse: minor discolouration, possible heating?
Palart.659	Limestone (pebble, fragment)	10.2 x 7.0 x 1.7	Obverse: Bison (1)	Obverse: potlidding, cracking on bottom edge
				Reverse: thermal fracture whole surface
Palart.660	Limestone (pebble, fragment)	13.0 x 7.5 x 3.8	Obverse: Bison (1)	Obverse: Black discolouration to top edge and parts of face
				Reverse: possible thermal spalling
Palart.661	Limestone (tabular plaque)	38.0 x 18.0 x 2.9	Obverse: Bison (2)	n/a
Palart.662	Limestone (tabular plaquette)	10.4 x 6.6 x 1.6	Obverse: Ibex (5)	n/a
Palart.663	Limestone (fragment)	10.4 x 7.0 x 2.4	Obverse: horse (1), wolf (1)	Reverse: limited localised black discolouration across the face
Palart.664	Limestone (pebble)	10.5 x 8.7 x 3.5	Obverse: Horse (1)	Reverse: possible rubefaction with black flecking, right edge
Palart.665	Limestone (tabular slab, fragment)	15.5 x 9.3 x 2.0	Obverse: Horse (1), indeterminate (2)	n/a
Palart.666	Limestone (fragment)	7.4 x 4.3 x 0.9	Obverse: Horse (1)	n/a
Palart.667	Limestone (thin plaquette)	17.5 x 12.0 x 2.7	Obverse: Horse (1)	Obverse: Rubefaction on left edge and bottom edge
				Reverse: rubefaction on right edge, bottom left edge, top left edge
Palart.668	Limestone (thicker slab)	13.3 x 10.8 x 4.75	Obverse: horse (1), ‘arrow’/‘dart’ (1)	Obverse: grey discolouration possibly linked to heating top edge
				Reverse: possible localised area of rubefaction and heat spall top left edge
Palart.669	Limestone (tabular fragment)	15.4 x 12.0 x 2.2	Obverse: Horse (1)	Obverse: Black traces on bottom edge and face
Palart.670	Limestone (block fragment)	9.2 x 5.2 x 2.2	Obverse: Horse (3)	Obverse: rubefaction bottom left corner
				Reverse: rubefaction bottom right corner
Palart.671	Limestone (pebble, fragment)	11.0 x 9.0 x 2.5	Obverse: bird (1/2)	Obverse: thermal fracture on bottom left edge, localised potlidding on the face
			Reverse: indeterminate	
				Reverse: cracking
Palart.672	Limestone (pebble)	22.0 x 15.0 x 5.0	Obverse: Chamois (3), horse (1)	n/a
Palart.673	Limestone (tabular, fragment)	5.4 x 3.2 x 0.7	Obverse: cervid (1)	n/a
Palart.674	Limestone (block)	13.6 x 12.0 x 5.0	Obverse: horse (2)	Obverse: rubefaction on right edge and face, some black traces
				Reverse: localised black traces on the face
Palart.675	Limestone (block)	10.1 x 7.5 x 4.0	Obverse: horse (2)	Obverse: rubefaction, bottom edge. Some black traces associated with rubefaction
				Reverse: rubefaction across the whole surface. Black traces.
Palart.676	Limestone (tabular, fragment)	11.0 x 7.0 x 4.1	Obverse: horse (1?), criss-cross lines	n/a
Palart.677	Limestone (tabular fragment)	9.4 x 6.4 x 1.0	Obverse: horse? (2)	n/a
			Reverse: Single parallel lines	
Palart.678	Limestone (pebble, flake)	7.5 x 5.4 x 0.9	Obverse: horse (2)	n/a
Palart.679	Limestone (pebble)	8.3 x 5.2 x 1.1	Obverse: bison? (1)	n/a
			Reverse: indeterminate	
Palart.680	Limestone (tabular fragment)	6.7 x 4.4 x 2.3	Obverse: horse (2)	n/a
Palart.681	Limestone (tabular block)	6.8 x 5.0 x 2.6	Obverse: horse (1), claviform? (1)	Obverse: possible rubefaction across the face
				Reverse: possible localised rubefaction left edge and face
Palart.682	Limestone (pentagonal fragment)	9.2 x 5.3 x 2.3	Obverse: Horse (1)	n/a
Palart.683	Limestone (tabular fragment)	7.1 x 5.0 x 1.3	Obverse: cervid (1)	Obverse: rubefaction across the bottom edge, extending to the middle of the face. Black traces. Possible thermal fracture of bottom edge
				Reverse: rubefaction to bottom edge
Palart.684	Limestone (pebble)	10.8 x 7.0 x 2.5	Obverse: Red deer stag (1)	n/a
			Reverse: Cervid (2), horse (2)	
Palart.685	Limestone (pebble)	14.7 x 12.5 x 3.2	Obverse: horse (6)	Reverse: cracking and potlidding across the face
			Reverse: curved lines	
Palart.686	Limestone (pebble)	13.0 x 11.0 x 2.0	Obverse: horse (1), bovid (1)	Obverse: rubefaction, top edge. Black traces on the edge
			Reverse: horse (1)	
Palart.687	Limestone (pebble)	13.7 x 13.0 x 2.4	Obverse: red deer (1), horse (1), cervid (2)	n/a
			Reverse: cervid (1)	
Palart.688	Limestone (pebble, fragment)	8.0 x 5.5 x 3.0	Obverse: ibex (1), horse (1)	n/a
Palart.689	Limestone (pebble, fragment)	8.5 x 6.5 x 1.2	Obverse reindeer (1), indeterminate lines	Obverse: Rubefaction, bottom edge. Black discolouration on bottom edge and face
				Reverse: possible rubefaction, top edge
Palart.690	Limestone (pentagonal fragment)	10.5 x 7.5 x 3.3	Obverse: reindeer (3)	n/a
Palart.691	Limestone (block, fragment)	16.3 x 10.5 x 2.4	Obverse: horse (3), reindeer (1)	n/a
			Reverse: reindeer (1)/indeterminate	
Palart.692	Limestone (pentagonal fragment)	14.8 x 13.0 x 2.1	Obverse: horse (1), reindeer (1)	n/a
Palart.693	Limestone (pebble, fragment)	16.5 x 5.6 x 3.7	Obverse: ibex (1), schematic lines	n/a
Palart.694	Limestone (pebble, fragment)	16.0 x 8.0 x 2.2	Obverse: anthropomorphic limbs (2) and torso (1), schematic lines	n/a
Palart.695	Limestone (block)	20 x 9.5 x 5.0	Obverse: horse (1), cervid (1), reindeer (1)	Obverse: Rubefaction on left edge and face
				Reverse: rubefaction to right edge
Palart.696	Limestone (fragment)	8.7 x 5.3 x 0.8	Obverse: Cervid (1), schematic lines	Reverse: possible thermal fracture
Palart.697	Limestone (fragment)	8.3 x 5.2 x 0.8	Obverse: cervid (1)	Reverse: cracking, possible thermal fracture
Palart.698	Limestone (fragment)	6.5 x 3.4 x 0.7	Obverse: horse? (1)	Obverse: rubefaction on right edge
			Reverse: indeterminate	Reverse: rubefaction on left edge
Palart.699	Limestone (fragment)	7.5 x 6.3 x 1.2	Obverse: indeterminate (1)	Obverse: rubefaction on top edge
				Reverse: rubefaction localised to top edge
Palart.700	Schist (fragment)	5.2 x 3.2 x 0.6	Obverse: indeterminate (1)	n/a
Palart.701	Limestone (pebble, fragment)	8.4 x 6.0 x 3.4	Obverse: horse? (1)	Obverse: rubefaction left edge
			Reverse: schematic lines / indeterminate	Reverse: rubefaction top edge
Palart.702	Schist (fragment)	6.0 x 1.8 x 0.15	Obverse: schematic lines	n/a
Palart.703	Limestone (pebble)	14.5 x 8.0 x 3.5	Obverse: schematic lines	Obverse: localised rubefaction with grey and black traces across the face
			Reverse: schematic lines	
Palart.704	Limestone (flake)	8.0 x 6.0 x 0.8	Obverse: schematic lines	n/a
Palart.705	Limestone (fragment)	12.8 x 8.2 x 1.2	Obverse: criss-cross diamonds/lattices	Obverse: rubefaction localised to a single edge
			Reverse: indeterminate	Reverse: rubefaction
Palart.706	Limestone (tabular block, fragment)	19.8 x 10.5 x 3.6	Obverse: schematic lines	Obverse: rubefaction, black traces to bottom edge
				Reverse: rubefaction, black traces to bottom edge
Palart.707	Limestone (pebble)	11.5 x 5.6 x 2.0	Obverse: schematic lines	Obverse: possible rubefaction with black traces to the right/bottom edge
Palart.708	Unknown geology (pebble, possible pendant)	8.3 x 1.3 x 0.7	Obverse: diagonal lines, partial perforation	n/a
			Edge: notches	
			Reverse: schematic lines, partial perforation	
Palart.709	Unknown geology (pebble, possible pendant)	5.1 x 1.7 x 0.9	Obverse: schematic lines	n/a
			Edge: notches	
n/a	Limestone	unknown	Obverse: horse (1)	Reverse: possible rubefaction across the face

Table showing a summary of plaquettes from Montastruc detailing the material from which they are made, type and quantity of engraving, and modifications caused by heat. Note that ‘black traces’ could link to residue of burned fuel, or possibly taphonomic manganese dioxide staining, and should be treated with greater caution. Information derived from [21, 60].

In total, the plaquettes feature 76 animal engravings across 44 supports, with superimpositions—animal forms overlying one another—being common [39] (Table 2). Animals depicted consist of horses (40), ibex (7), reindeer (7), red deer (6), bison (5 or 6), chamois (3), anthropomorphic forms (1 or 2), bovid (1), birds (1), and wolf (1) [21]. Naturalistic depiction is common, reflecting attention to the anatomy and seasonal appearance of animals, though some variation in execution is evident, possibly reflecting differences in skill [39, 52] or artistic choice. The plaquettes have been suggested to represent an *in situ* deposit based on the homogeneity of their engravings [21]. In some cases, recent damage—white in colour—is evident, likely caused by tools during excavation or from subsequent transport. This provides an insight into the colour of fresh engravings made with stone tools during the Magdalenian.

Recent analysis by Needham [60] on plaquettes from the de l’Isle collection integrated 3D modelling using a MechScan™ 3D structured light macro scanner alongside use of a hand lens (x6), low power (x10—x20) binocular microscopy with cold light sources mounted on swan neck attachments, and photography using a Nikon D5500 camera with AF-S VR MICRO-NIKOR 105mm F/2.8G IF-ED macro lens mounted to a copy stand, to develop new insights into these objects despite the limited archaeological context. 3D capture techniques have previously been used successfully to consider Palaeolithic parietal art production and its relationship to the cave wall [65–72], albeit with some limitations [73]. It has seen increasing application to portable art including plaquettes [3, 19, 74–77], offering a means to understand engraving in relation to natural features of the support on which it was made. New analyses [60] confirmed earlier reported findings [13, 78] regarding the engravings present on each plaquette, and revealed new insights about the patterns of heating and placement of engravings on surfaces.

**Fig 1:**
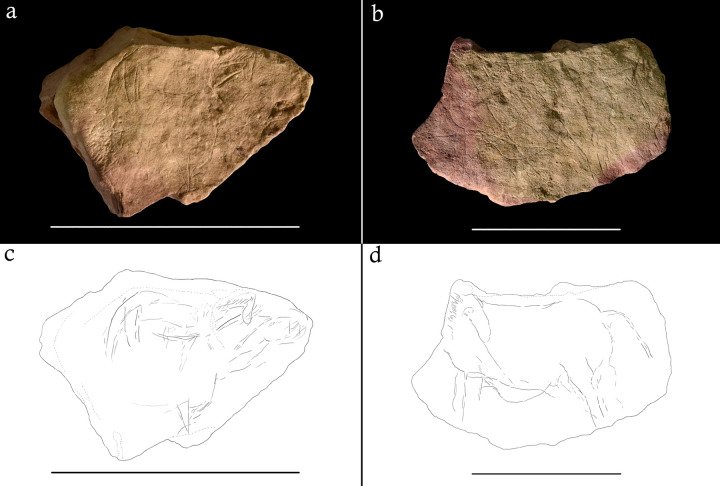
Photographs and digital tracings of plaquettes 675 (a; c) and 667 (b; d) from Montastruc. Scale bar below each plaquette is 10cm in length.

The Montastruc plaquettes evidence a pattern of heating after engraving in some cases, with bands of pink discolouration (henceforth rubefaction), cracking, thermal fractures, and pot lids (Fig 1; Table 2). 27 (50.94%) plaquettes show evidence of heating and burning and 5 (9.43%) evidence fragmentation [60]. This pattern is not evident on the four non-limestone plaquettes at the site and neither does the organic art assemblage show heating traces, raising the possibility that the association of limestone plaquettes to combustion activities is exclusive at Montastruc [60]. The placement of engravings appears sensitive to the form of the limestone, sometimes incorporating natural features such as block shape, cracks, and undulations (Fig 1). Where engravings on plaquettes were superimposed, a similar approach was adopted: rather than ignoring or engraving over previous depictions, animals were often melded together or fitted around each other, and sometimes body parts were recycled. Plaquettes 675 and 667 clearly demonstrate these features of the Montastruc plaquettes. Plaquette 675 (Fig 1A) shows a horse (right profile) and bovid (right profile), with rubefaction to the bottom edge. The horse is fitted to the top edge and uses a natural crack to form the projecting front leg. Melding of animal forms is evident: the abdomen and neck of the horse form the back and neck of the bovid, while the head of the horse forms the ear of the bovid. Plaquette 667 (Fig 1B) shows a horse (left profile) with the form fitted around the angular protrusions to the top left edge inspiring a head turned back on itself. Rubefaction is visible to the left edge and bottom edge.

The de l’Isle collection from Montastruc is characteristic of many stone plaquette assemblages; by virtue of their excavation in the mid-19th to mid-20th Century, the original context of many plaquette assemblages is limited or unknown, leading to a focus on the engraved depictions. However, in the case of Montastruc, the presence of heating traces on some plaquettes may be one means of providing insight into their context of use, when integrated alongside an analysis of engraved forms. A diverse toolset is proposed to achieve this, integrating material insights from the limestone itself with a programme of microscopy, 3D modelling, DStretch©, VR modelling and experimental archaeology.

## Why heat limestone? formulating hypotheses

The heating traces on the Montastruc limestone plaquettes, a feature unique amongst art objects at the site, invites a deeper consideration of the material properties of limestone and whether these can provide insights into use. Limestone forms in warm, marine conditions as biological detritus accumulates, occasionally forming fossils [79], and often includes impurities such as clay, silica, magnesium, manganese and iron [80–82]. It is the iron impurities that react during heating, causing dramatic but predictable colour changes, revealing pink, red, and grey hues [83–85]. Areas of rubefaction visible on the Montastruc limestone plaquettes appear to be a consistent feature of heating limestone to a specific temperature threshold. This has been observed in other Upper Palaeolithic contexts, such as at Chauvet cave where rubefaction on the limestone walls was caused by heating the surface above a temperature threshold of approximately 250–300°C, which changes to a grey hue during more intensive burning at temperatures above 350°C [86–89]. These temperature thresholds are consistent with observations from fire-damaged limestone buildings [90, 91] and experimental contexts that heated limestones of different geologies [92–95] (Table 2). The presence of heating on the Montastruc plaquettes appears to be indicative of these objects being exposed to relatively high temperatures, likely due to direct contact with fire. However, the specific conditions or activities that may have caused this are difficult to ascertain from an analysis of the limestone material alone.

Whilst the lack of heating on other organic objects and non-limestone plaquettes from Montastruc alludes to taphonomic action likely not being the cause of heating traces on the limestone plaquettes, this cannot be ruled out completely. Incidental taphonomic factors have been previously proposed as a potential explanation for heating traces on limestone plaquettes at other sites [33], and may be the case for the Montastruc plaquettes. This possibility constitutes the first hypothesis to be explored in this study: **Hypothesis 1) heating traces on the Montastruc plaquettes were caused by incidental taphonomic action.**

It is also possible that limestone plaquettes may have been utilised for other activities by virtue of their size, shape, and material, suggesting a separation between the artistic production and use of engraved plaquettes, and their subsequent use in heating activities. The use of stones in association with fire has been suggested to be a common occurrence in the Magdalenian [99] (Dumaràay and Caron 2010). The presence of fire-cracked rock (FCR) in layer IV at Montastruc, recorded by Bétirac (see Table 1) may suggest undecorated limestone blocks were utilised for functional activities at the site, with the decorated plaquettes possibly implicated in these activities at a later stage in their use-lives. Limestone is suitable for a variety of activities related to heating due to its ability to effectively transfer and radiate heat. In the Magdalenian, several archaeological contexts demonstrate that decorated plaquettes were used alongside undecorated limestone blocks as part of the fabric of a hearth, such as at Étiolles [45] and Labastide [46, 51]. It has been suggested that they were used to construct hearth structures at Monruz and Champréveyres (Switzerland) [100–102]. Work by Amy [103] supports the use of limestone blocks in this way, showing that this can produce a fuel-efficient hearth; an important consideration in the Magdalenian where temperatures were cold [100] and woody fuel materials were scarce [101, 102]. In addition to their direct implication in hearth construction, plaquettes may have also been heated for other functional activities. For example, similar stones in pre-Columbian North American contexts were utilised as boiling stones for cooking and sanitisation purposes, and this has been suggested as a potential use for stones that evidence heating in the Upper Palaeolithic [104–107]. Whilst the use of limestone for this purpose is rare—heated limestone submerged in water produces a hydrated lime slurry unsuitable for consumption [105]—heated water can be useful for other non-consumption related activities [108, 109]. It is possible, therefore, that the limestone plaquettes at Montastruc were implicated in functional heating activities and selected for their material properties and morphology. This possibility constitutes the second hypothesis to be explored in this study: **Hypothesis 2) heating traces on the Montastruc plaquettes were caused by functional activities, unrelated to the engraved forms.**

The presence of heating traces on the Montastruc plaquettes may have been directly related to the engraved forms, with the visual effects of heating plaquettes adding an experiential quality to the art. Limestone undergoes dramatic physical changes when heated, exhibiting vivid changes in colour and thermal fracturing or breakage at higher temperatures, which may have been attractive material properties to Magdalenian artists at Montastruc. The intentional heating and thermal fracturing of decorated plaquettes has been previously argued to be an important feature of their use, as a means to ‘desacralize’ plaquettes [47, 51]. Indeed, at sites such as La Marche and Labastide, plaquettes appear closely associated with hearths, but for no apparent functional reason [51]. The dramatic effects of heating portable art objects has been recognised in other Upper Palaeolithic contexts, such as the exploding loess figurines reported from the Gravettian site of Dolni Vĕstonice (Czech Republic) [110–112]. In addition, the effect of a flickering light source on the undulating topography of limestone has been previously argued to be an integral feature of some parietal cave art, adding dynamism to the depicted animal forms [113–118]. The lighting of parietal cave art in this way has been recognised at Chauvet to produce similar rubefaction traces on the limestone surface [86, 87, 89]. These possibilities constitute the third hypothesis to be explored in this study: **Hypothesis 3) heating traces on the Montastruc plaquettes were caused by non-functional activities related to the engraved forms.**

## Materials and methods

An experimental programme was designed to test these hypotheses, informed by an appreciation of the chemical and physical changes that occur in limestone when heated (Table 2) and published theories about the association of limestone plaquettes to heating activities recorded at other Upper Palaeolithic sites. The use of experimental archaeology in the analysis of questions related to Palaeolithic art is not without precedent [87, 89, 119, 120] and is well suited to addressing artefact use in situations where the archaeological context is insufficient to resolve the question directly.

In total, five experiments were designed and executed at the York Experimental Archaeology Research (YEAR) Centre, Department of Archaeology, University of York. Taphonomic hypotheses were tested through (A) placing completely buried, partially buried, and exposed plaquettes at different distances away from a hearth; functional uses were tested through (B) use as boiling stones and (C) use as components of an open oven structure; non-functional activities were tested through (D) recording the visual effects of pouring water on heated plaquettes and (E) placing engraved plaquettes in spatial proximity to a hearth at night. Temperature data were recorded for the limestone plaquettes at regular intervals for experiments A, D, and E and before and after exposure to water for experiments B and C (see S1 File), to determine the temperature ranges for which thermal modifications were visible on the limestone. Combined, the experiments enabled a reference collection of heating and burning signatures on replica limestone plaquettes associated with different combustion activities to be built. This was compared against the archaeological examples from Montastruc as a means of providing insights into their use. The experimental protocols used are not without limitations: the experiments were actualistic, with varying environmental conditions, refuelling regimes, and changing fire morphology. Replica limestone plaquettes were produced using reclaimed limestone with unknown geological origin, and therefore were not an exact match for Montastruc, both in terms of size and chemistry. The engraved motifs on the replica examples did not directly emulate those from the Montastruc plaquettes. However, limestone of different geologies acts in predictable ways when exposed to heat (Table 3) and the experimental data is used primarily to inform the VR models, which simulate conditions on the archaeological specimens directly. A full description of the protocols used in each experiment is detailed in the S1 File.

**Table 3:** 

0–150°C →	100–300°C →	200–400°C →	400–600°C →	>600°C →	700–800°C →
Limestone loses water, goethite begins to rearrange to haematite	Goethite transformation, 250–260°C dehydroxylation of goethite	Hydroxyl group loss and dehydroxylation of clays, mass decreases, brittleness increases	Full oxidation of iron ions begins and completes at 600°C	Lime formation begins to occur superficially on the limestone	Total mechanical failure of the limestone, wide and irregular fractures, pulverisation occurs
Natural colour	Colour change to pink	Deeper pink colour	Colour change from pink to grey	Grey colouration, white lime deposits on surface	More developed grey colouration, white lime deposits throughout

Table showing the changing material properties of limestone with increasing temperature. Observations derived from the following sources [86, 87, 89–98]. Overlaps in temperature ranges across categories reflect the observations presented by the papers cited, which record limestone in different circumstances, such as fires in buildings or controlled experiments where a steady temperature was maintained. The table therefore communicates a trajectory broadly applicable to any limestone undergoing heating but idiosyncrasies are to be expected when heating different limestones under varying actualistic conditions.

**Fig 2:**
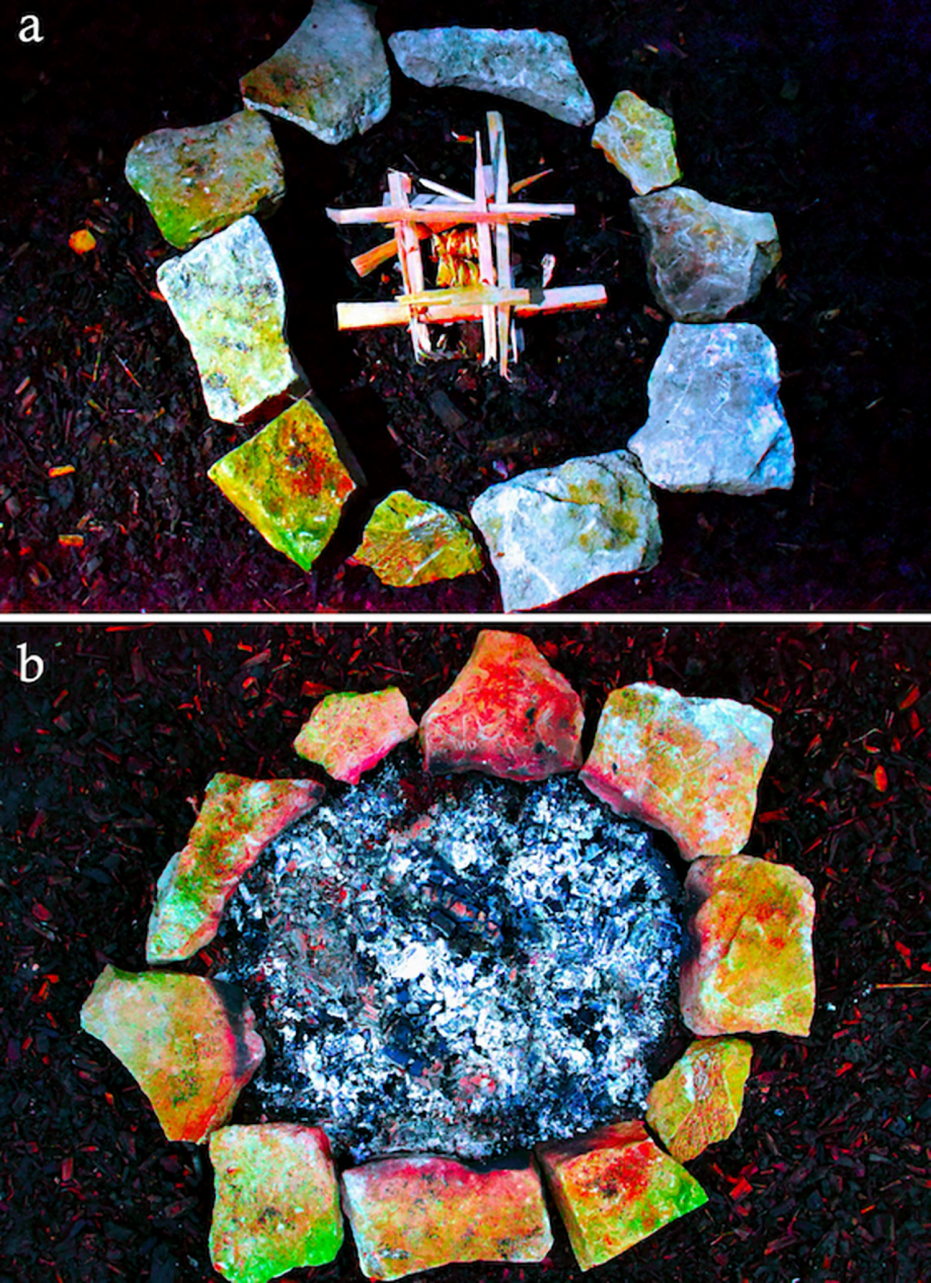
DStretch manipulated images in the LAB colourspace showing the difference between DStretch images of replica limestone plaquettes in experiment E (A) before heating and (B) after heating. Note that some reddish hues are visible in the DStretch image of replicas before heating, that may be caused by adhering sediment or the natural colouration of the limestone. Thus, the use of DStretch alone is not reliable to identify rubefaction traces.

Specific comparison between the archaeological and experimental plaquettes was facilitated through colour enhancement of high resolution photographs via DStretch©. DStretch© is a plugin for the ImageJ© digital imaging software first developed for use in rock art research by John Harman in 2005, and enables the user to manipulate specific colours in an image [121]. In this case, it was used to visualise the rubefaction and grey discolouration caused by heating on both the experimental and archaeological plaquettes by manipulating the LAB colourspace matrix. DStretch© has been used to good effect in archaeology to visualise and identify faded rock art depictions [121–125], but not to visualise rubefaction caused by thermal modification. DStretch© augmented observations that were made with the naked eye by visualising colour changes partially obscured by overlying soot deposits and enhancing subtle discolouration patterns derived from experiments where exposure times or temperatures were low. Use of DStretch© to demarcate thermal modification does, however, have limitations: while it can visualise pink/red/grey hues where present, it cannot discern whether the source is derived from the stone itself, heating, pigment or sediment (Fig 2). Thus, it is best utilised, as in this paper, to augment established micro- and macroscopic techniques in analysing heating traces.

Experimental and DStretch© results were used to inform a series of VR lighting simulations built using the 3D models of the Montastruc plaquettes produced by Needham [60], to capture the visual effects of exposure to the roving light cast by a hearth. Models were first reduced within Agisoft Metashape to lower the face count of the mesh and the overall file size, allowing the models to be imported into other softwares. In Substance Painter, reduced models were recoloured to make the engraved lines white, simulating fresh engraving. Deeper engravings were recoloured with a thicker line and shallower engravings were recoloured with a thinner line, to capture any visual distinction between variation in the depth of engraving in the lighting simulation. The recoloured 3D models were imported into Unity, an open source virtual reality gaming software, rescaled to their original size, and placed in proximity to a virtual hearth. The virtual hearth was given an arbitrary footprint of 0.5m in diameter and a warm flickering light source of approximately 1900K that cast light across a radius of 2.5m, consistent with published experimental observations of low lumen light sources [126], emulating the light cast from a small hearth. Placement of the Montastruc plaquette 3D models was determined by the specific heating evidence on each plaquette, with the area discoloured by heating placed in closest proximity to the virtual hearth. This choice was also informed by experimental results (see S6 File). The visual effects of the light source on 3D models of the Montastruc plaquettes were recorded as a screen captured video (see S2–S6 Files).

## Results

### Heating traces: Experimental and DStretch© results

The experimental results confirmed previous archaeological and experimental observations that limestone blocks and plaquettes can be used effectively for a variety of functions in combination with fire, including as cooking stones, as hearth furniture or structures to create a fuel efficient fire, as a source of heat storage, or potentially to heat water. To an extent, these different activities produce diagnostic changes in the limestone, including thermal fracturing and colour change (see S1 File for full results by experiment). The experimental results confirm a close association between colour changes and temperature thresholds: rubefaction suggests a threshold of around c.100-300°C was reached, while grey discolouration suggests a more intense heat, c.600°C or more. The presence of soot suggests temperatures below 400°C [127]. Predictably, the location and extent of colour change correlates with proximity to heat source: a plaquette positioned on the edge of a hearth will likely show discoloration favouring the proximate edge and face; a plaquette positioned within the centre of a hearth shows intense colour change on most surfaces. This data enables insights into their spatial positioning and association with hearth structures, providing insight into how Magdalenian stone plaquettes were used.

**Fig 3:**
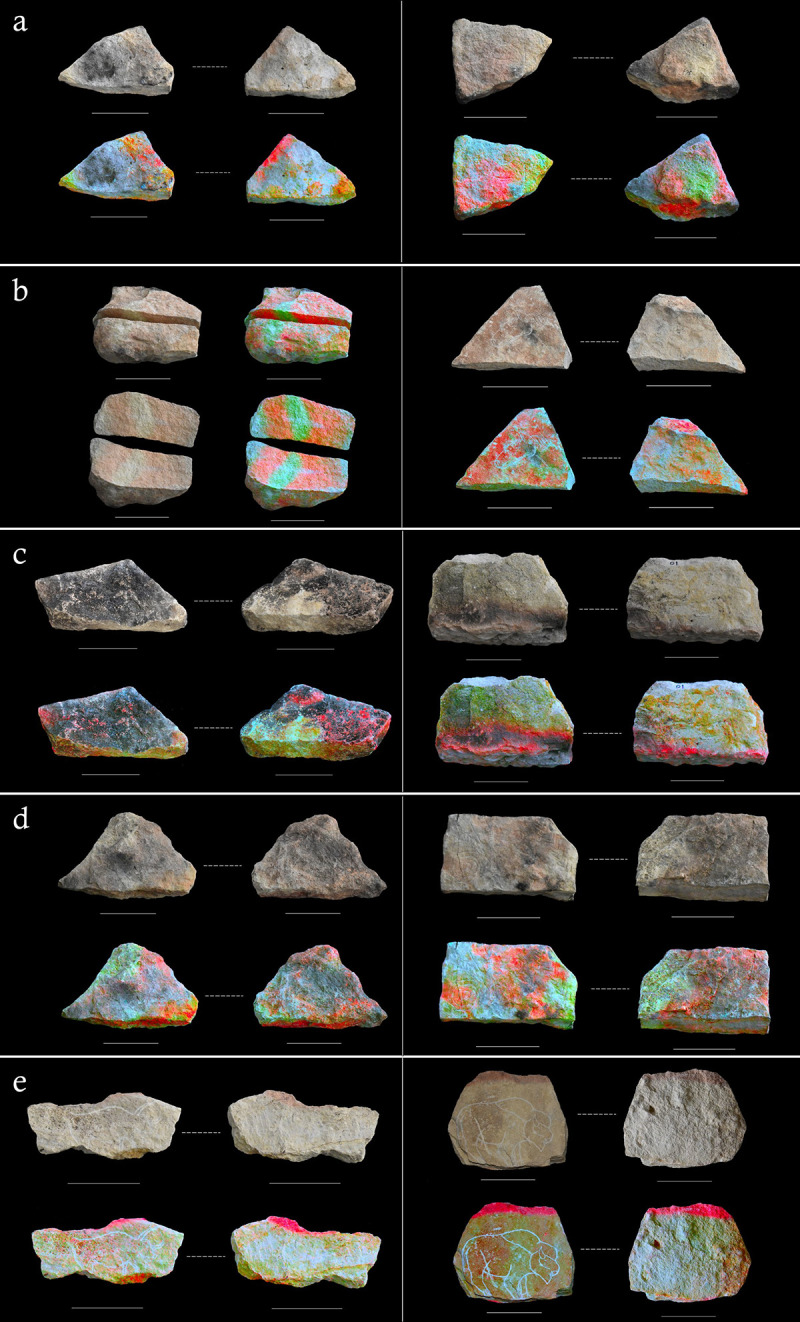
High-resolution photographs and DStretch© images of example replica plaquettes from each experiment. (A) shows plaquettes from the taphonomy experiment that were in direct context with the fire, placed on the surface both uncovered (SU1: left) and covered with sediment (SC1: right). (B) shows replica plaquettes PB3 (left) and PB4 (right) from the boiling stone experiment. (C) shows replica plaquettes A3 (left) and D1 (right) from the hearth oven structure experiment, (D) shows replica plaquettes WP1 (left) and WP3 (right) from the water pour experiment. (E) shows replica plaquettes FS1.1 (left) and FS1.2 (right) from the fireside night experiment. See the S1 File for a full description of the results from each experiment. Scale bar below each replica plaquette is 8cm in length.

**Fig 4:**
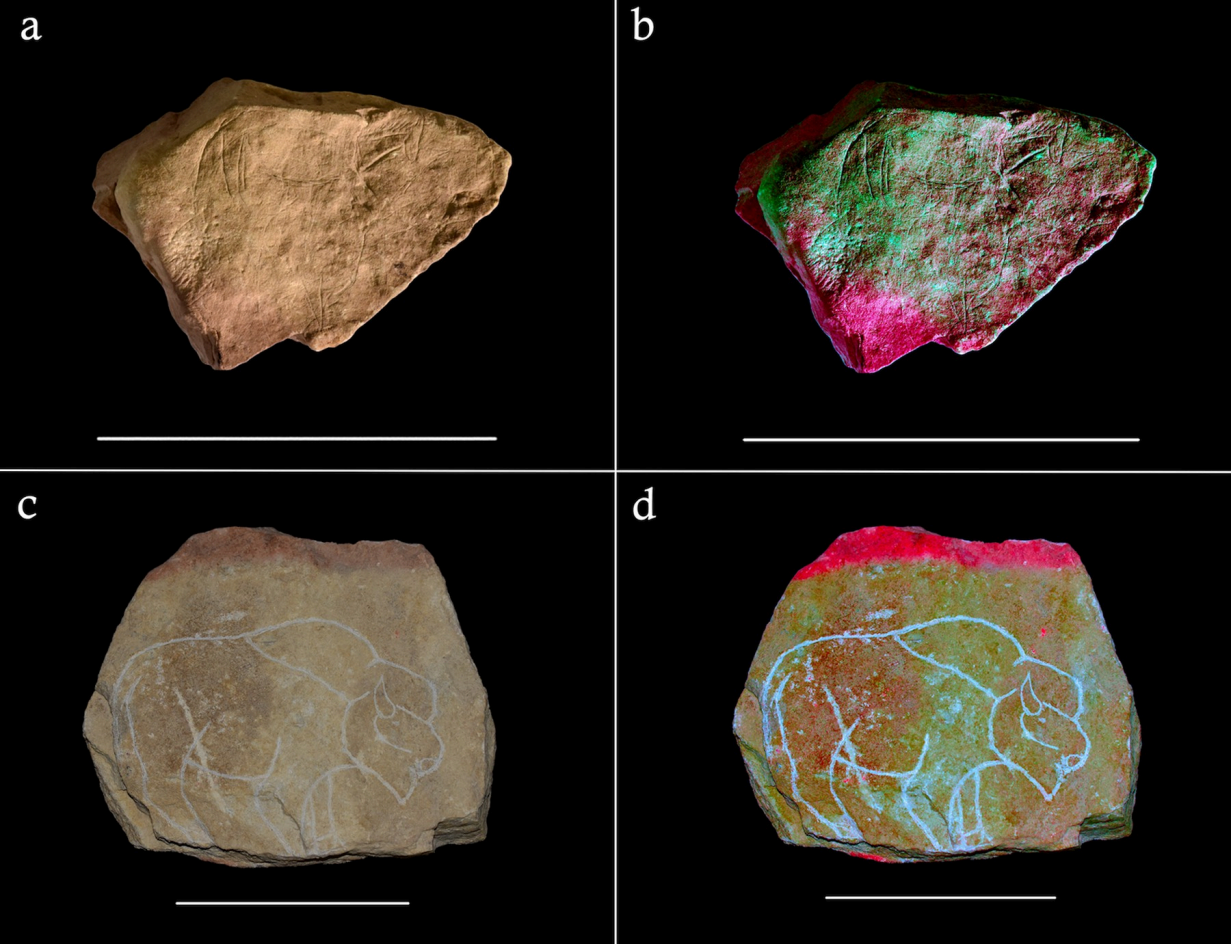
Photographs showing a comparison between Montastruc plaquette (a, b) and replica plaquette FS1.2 (c, d) used in experiment E. b and d are DStretch© manipulated images using the LAB colourspace matrix to enhance pink discoloration caused by heating. Scale bar below each plaquette is 10cm in length.

**Fig 5:**
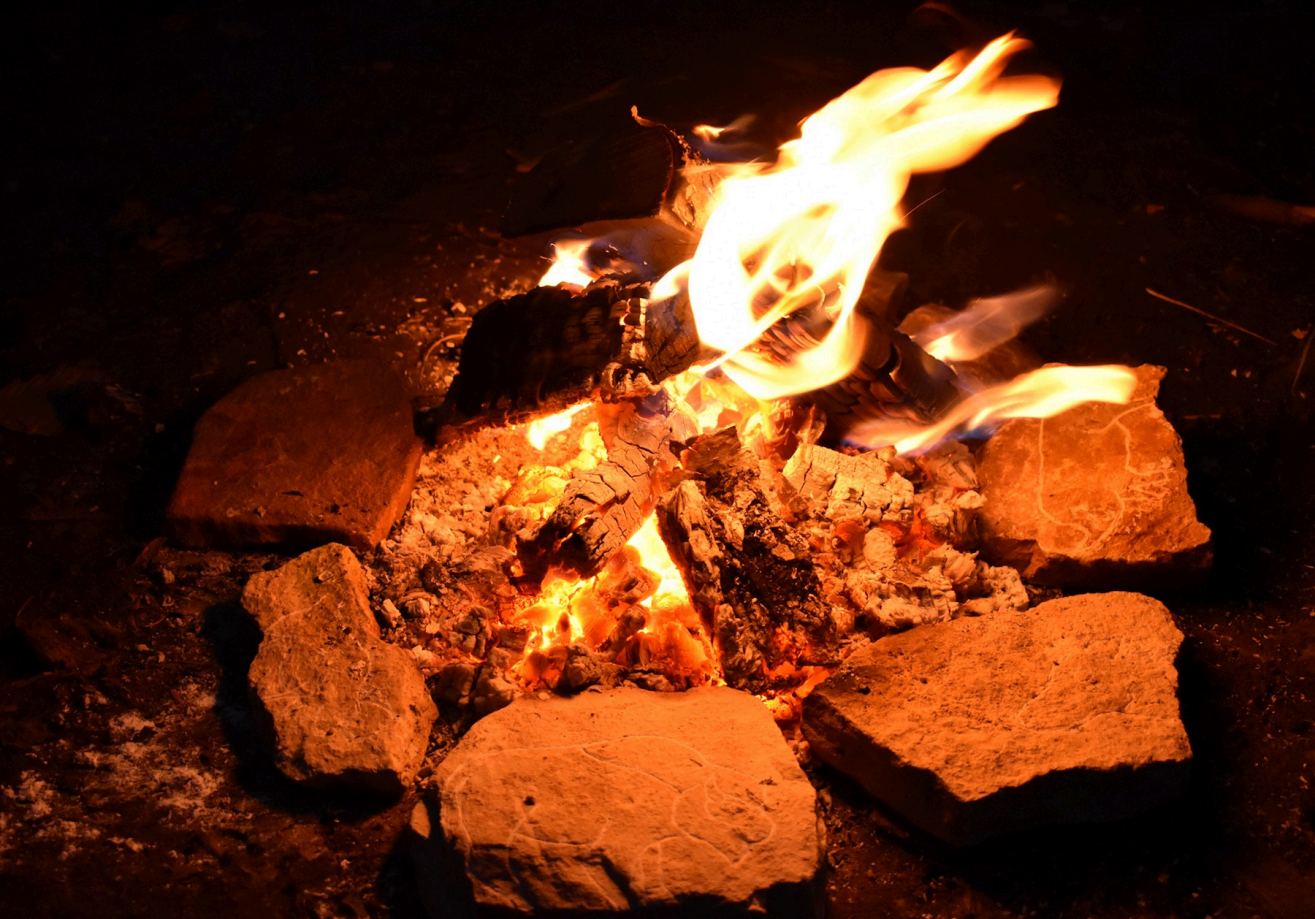
Photograph showing ambient light levels and the position of replica plaquettes in relation to the fire during experiment E.

Images manipulated via DStretch© images were used to facilitate a comparison between the heating pattern observed on the experimentally-produced replicas and plaquettes from Montastruc (Figs 3 and 4). Some of the Montastruc plaquettes appear to closely match the diagnostic heating pattern generated on replicas from experiment E, which were placed in a circular formation in close proximity to each other and a small central fire (Figs 2 and 5). In both rounds of this experiment, variation in the extent of discolouration of replica plaquettes within the same hearth feature was observed. This appeared to be, in part, linked to ambient conditions, such as wind direction. The replicas downwind showed clearer signs of discolouration on surfaces proximate to the heat source, whilst those further away or upwind only showed this discolouration on the edges in direct contact with the fire, if at all, creating a distinctive pattern across the assemblage. Temperature data obtained from the second round of experiment E (FS2) supported this observation. A replica plaquette (FS2.1) placed downwind reached temperatures up to 544°C for the edge orientated closest to the fire, whilst replica plaquette FS2.3 placed upwind only reached a maximum temperature of 70.6°C. This pattern is consistent with the archaeological specimens from Montastruc which, based on their similarity in style of engraving, were argued to have been deposited *in situ* [13], yet not all show evidence of heating.

The experimental data suggests that it is possible that plaquettes from Montastruc with no visible evidence of heating may have still been heated, but failed to meet the temperature threshold of c.100-300°C to produce rubefaction. Experimental replicas that did not reach this threshold exhibited no evidence of heating traces, meaning their association to a hearth in this configuration would be archaeologically invisible, without contextual spatial information. Examples with no visible heating traces at Montastruc may possibly have been part of the same or different combustion episodes as those with visible heating traces. Additionally, in some cases plaquettes at Montastruc may have been heated for protracted, repeated or more intense burning episodes, causing thermal fractures and/or pot lidding. While the experimental data does not entirely rule out other contributing factors, such as taphonomy, when taken alongside the lack of heating evidence on organic portable art and non-limestone engraved plaquettes at the site [60], anthropogenic non-functional action seems the more likely cause in this case.

### Visual effects: Experimental and virtual reality results

The placement of experimentally-produced plaquettes in proximity to a fire resulted in notable visual effects. As the light cast from the hearth hit the replica plaquettes at an oblique angle, it appeared to the naked eye to emphasise the relationship between morphological features of the limestone support and engraved depictions. The fire light also added dynamism to the engravings as it flickered across the engraved surface.

**Fig 6:**
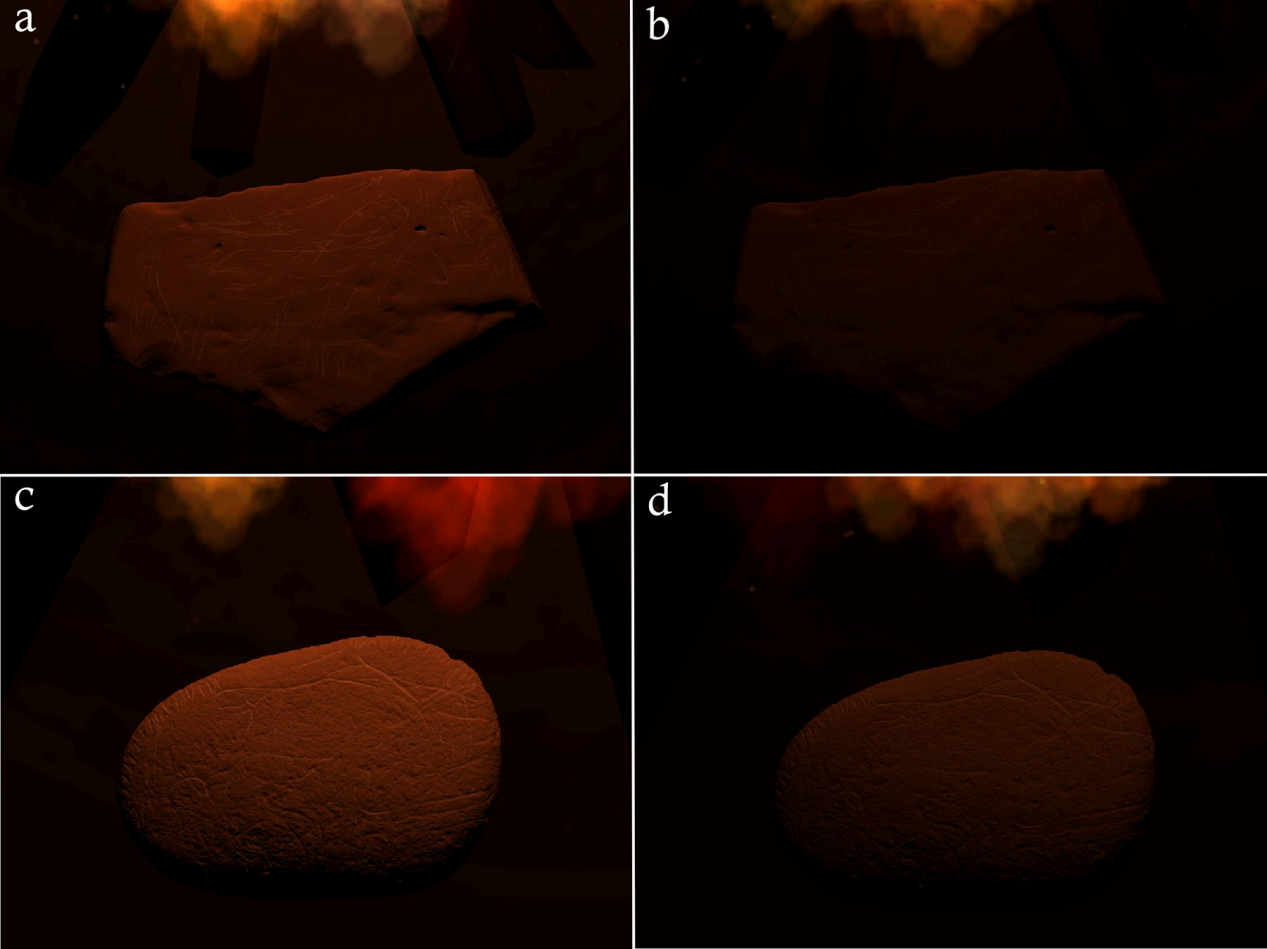
High resolution screen captures of the 3D models of plaquette 691 (a, b) and plaquette 684 (c, d) from Montastruc in the VR simulation at the maximum and minimum illumination as the light flickered. For both plaquettes, aspects become more or less visible as the light shifts, adding to a sense of movement.

VR modelling was used to simulate these visual effects using 3D models of the Montastruc plaquettes, so they might be considered directly in a similar lighting condition and spatial configuration as used in experiment E, and to corroborate the qualitative observations made during the experiment. The real-time visual effects of the dynamic, low lumen light source in the VR simulations reveal how the engraved surfaces of the Montastruc plaquettes similarly come to life under such conditions: the engravings appear more animated and dynamic, despite being static. This visual effect was particularly notable for superimposed engravings, with the roving light source serving to illuminate aspects of different engraved forms unpredictably, enhancing the sense of movement and dynamism. The VR simulation of plaquette 691 from Montastruc clearly captures this effect: the flickering light source draws focus of one engraved horse form and then another, giving the impression that the figures are moving across the plaquette’s surface (Fig 6; see S2 File for VR simulation video). The superimposition of forms may have been an intentional feature of the engravings with the artists intending to capture animation, as is seen elsewhere in Magdalenian portable and parietal art [113, 114, 128]. This visual experience evokes a sense of narrative embedded into observing plaquettes, which varies depending on the engraved form and light conditions. For example, plaquette 684 has multiple species superimposed in varying positions on the surface which, when exposed to a roving light source, resulted in different animals shifting in and out of view (Fig 6; see S3 File for VR simulation video). It is possible this was a desired effect of engraved plaquettes at Montastruc.

**Fig 7:**
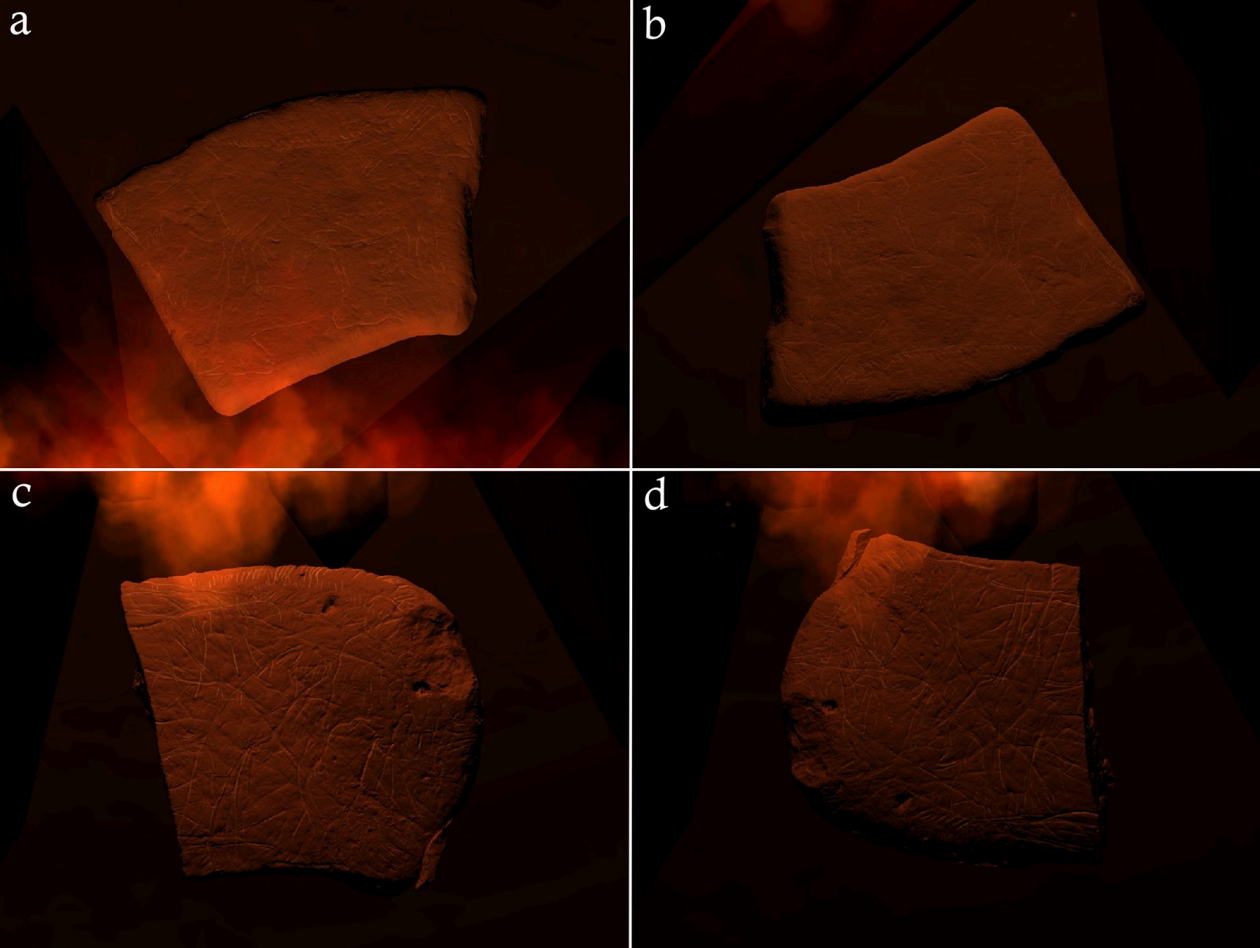
High resolution screen captures of the 3D models of plaquettes 662 (a, b) and 685 (c, d) from Montastruc in the VR lighting simulation. The different position of the viewer or orientation of the plaquette renders visible or ambiguates different figurative depictions on the plaquette. For plaquette 662 one orientation reveals a large ibex (a), and another reveals two ibexes facing each other (b). For plaquette 685, several horse heads can be perceived in one orientation (c) whilst a grazing horse can be seen in another (d).

The VR simulations drew attention to another visual effect, which was made particularly clear for plaquette examples where animal engravings are present in different orientations. When plaquettes were observed in VR with the camera set at different positions, certain figures appeared to become visible and distinct, whilst others were rendered ambiguous. For example, the varying orientations of ibex depictions on plaquette 662 meant that as the plaquette was viewed from different positions around the hearth, certain ibex would become distinct to the viewer whilst others would appear ambiguous (Fig 7; see S4 and S5 Files for VR simulation video). A similar effect was observed for plaquette 685 where the superimposed horse engravings appeared variously distinct or ambiguous depending on viewer position (Fig 7; see S6 and S7 Files for VR simulation video). It is possible, therefore, that the orientation of a plaquette next to a hearth, the viewing position and perhaps the movement of viewers in relation to the hearth were important considerations in the observation of plaquettes under these conditions.

## Discussion: Art by firelight?

Results from the experimental and digital techniques have provided new insights into the potential use of Montastruc plaquettes, and their close association with hearths. Although the results cannot completely dismiss the possibility of heating traces being caused by other activities, such as taphonomic action, the heating evidence present on a number of the Montastruc plaquettes appears to be more likely the result of their intentional placement proximate to a fire. The lack of heating traces on non-limestone engraved plaquettes and organic art may further support this interpretation. The VR simulations suggest that the dynamic light conditions of this configuration of plaquettes around a fire reinforce the blurring of natural and cultural features evident on the engraved surfaces. Plaquette use at Montastruc can be seen to have had functional and non-functional aspects. Plaquettes placed in proximity to a hearth also serve to contain the footprint of the hearth, but this could be as readily achieved with unmodified limestone as engraved limestone. The careful placement of the limestone to surround and contain the hearth seems to have carried deeper meaning, possibly both in the colour changes this encouraged in the limestone, but also the dramatic effect the ever-shifting light from the fire had on the engraved limestone surfaces.

These insights invite a broader discussion surrounding the use of plaquettes at Montastruc, informed by aspects of Magdalenian art production more widely. The sense of dynamism and movement created by exposing the Montastruc plaquettes to a roving, low lumen light is evocative of similar lighting conditions experienced when viewing Palaeolithic art created in cave environments. Cave art spaces necessitated a mastery of dynamic elements within their immediate contexts: darkness [116–118]; light [126]; art placement and space [117, 125, 129–132]; perhaps also sound [133–142]; and the fitting of forms to a morphologically variable and complex surface [116, 143–146]. Conceptual linkages can possibly be drawn between the negotiation of these elements in cave art and the plaquettes at Montastruc. While the scale and setting differ, some of the artistic choices that were negotiated resonate.

In both cases—art within caves and Montastruc plaquettes experienced by firelight—the commingling of complex surfaces, engraved forms, and shifting light may have had a visceral effect on the observer. These conditions, particularly moving light and shadows, are conducive to triggering visual and perceptual psychological responses. The visual system is predisposed to use shadows and lighting to understand the depth and dimensions of an object [147]; shifting light across a surface, therefore, can create the illusion that an object may be moving in depth, even if it is static in size and position [148–153]. The sensitivity to pre-existing forms in the Montastruc plaquettes may allude to another psychological response being triggered: pareidolia—perceiving recognisable forms within random patterns—which may be more potent under low light conditions [115, 145, 154]. This is a typical feature of human neurology [155, 156] and is shaped by a person’s experience and visual expertise [156]. For example, Harel [157] found that birdwatchers can efficiently identify individual birds to the same level that most people in the Western world discriminate between individual faces. Magdalenian people were likely visual experts in recognising animal forms [154, 158], making this a theme likely to feature prominently in their pareidolic experiences, triggered by working with morphologically complex rock surfaces in low light levels. The susceptibility of human neurology to pareidolia and its universal nature suggests this may have been a shared social experience for plaquettes, in contrast to the discrete and exclusive experience of altered states of consciousness induced by trance proposed for other forms of Palaeolithic art [159–164]. Indeed, hearths can be social settings [165] and the presence of plaquettes alongside objects used in daily life at Montastruc indicates they may have been used in these social contexts by the fireside.

The results reveal new insights into the use of plaquettes from Montastruc, suggesting they were engraved and positioned by hearths where properties of the limestone material, the engraved forms, and firelight entwined to create a visceral visual experience within what may have been a rich and active nighttime socio-cultural setting [165]. It is possible that the making and experience of plaquettes at Montastruc may have been a means through which relationships with animals were negotiated. Limestone may have been particularly suitable in this role due to its often evocative shape, which under raking light conditions might have triggered pareidolic experiences, bringing to mind particular animal forms.

## Conclusion

An approach utilising experimental archaeology alongside novel digital techniques has allowed for the association of the Montastruc plaquettes with fire to be tested. Results suggest that the plaquettes were placed in proximity to a hearth, based on the similarity of heating modifications between experimentally produced replicas positioned around a hearth setting and archaeological results when compared using DStretch©. Observations noted during the experiments suggested the placement of plaquettes in this configuration at night may have had dramatic visual effects, emphasising the material properties of the limestone and the relationship between engraved forms and the support morphology. Virtual Reality was utilised to explore these visual effects for 3D models of the Montastruc plaquettes, and suggested that under a dynamic low lumen light source the engraved forms appeared animated. The integration of natural features of limestone and the animation of depicted forms under firelight closely parallels some parietal art. This perhaps indicates this behaviour was important to Magdalenian artists as a means of negotiating relationships with animals in their world.

The application of established (micro- and macroscopic analyses, experimental archaeology, DStretch, 3D modelling) and new (VR modelling) techniques has facilitated a new interpretation of the contexts of production and use of limestone plaquettes at Montastruc, a site with limited archaeological context. There is potential for these or similar techniques to be applied successfully to other collections—whether plaquettes or other artefact types—with similar limitations in their archaeological context. More work can be done to develop the approach and refine the results. Greater precision in the analysis of heating traces may be achieved through performing chemical analysis of the Montastruc limestone plaquettes and matching this to material collected from the region for repeat experiments. Recording lux and continuous temperature data using thermocouples during the experiments may allow for greater insight into hearth configuration, the temperatures generated and the difference to plaquette edge temperatures, and light values. Using ColorChecker may further allow for the colour intensity of rubefaction to be associated to particular temperature thresholds, where the limestone geology is the same for experimental and archaeological examples. These approaches would be particularly appropriate when working with collections with more robust archaeological context where hearth size and configuration can be modelled with greater precision. Additionally, simulating lighting conditions in VR for parietal art in cave environments may allow for further comparisons to be drawn between these two types of art. Nevertheless, the techniques and results presented in this paper demonstrate the potential of digital and experimental approaches in yielding new insights for objects with limited archaeological context.

## Supporting information

S1 File Appendix containing experimental archaeological protocols and results. (PDF)

S2 File Video of plaquette 691 in the VR lighting simulation. (MP4)

S3 File Video of plaquette 691 in the VR lighting simulation. (MP4)

S4 File Video of plaquette 662 in the VR lighting simulation. (MP4)

S5 File Video of plaquette 662 in the VR lighting simulation, orientated 180 degrees. (MP4)

S6 File Video of plaquette 685 in the VR lighting simulation. (MP4)

S7 File Video of plaquette 685 in the VR lighting simulation, orientated 180 degrees. (MP4)

S8 File Video of experiment E, demonstrating the visual effects of plaquettes under firelight. (MP4)
