# Supplementary material for: Art by firelight? Using experimental and digital techniques to explore Magdalenian engraved plaquette use at Montastruc (France)
Source: PLoS One. 2022 Apr 20;17(4):e0266146. doi: 10.1371/journal.pone.0266146 (PMC9020732; doi:10.1371/journal.pone.0266146)
Supplement: S1 File — (PDF) [file pone.0266146.s001.pdf]

Supplementary Information

Appendix: Experimental protocols and results

General experimental protocols

Experiments A-E were carried out at the York Experimental Archaeology Research (YEAR) Centre, University of York, during winter with ambient temperature ranging between 0-5°C. Replica plaquettes were produced for each of the experiments from pieces of reclaimed limestone, engraved using replica flint burins. Chemical analysis of the Montastruc plaquettes was not possible and so the similarity of composition between experimental and archaeological limestones was not testable. However, the limestone selected was of a similar texture, size, and shape. Initial testing showed that heating revealed similar colours to those seen on the Montastruc plaquettes, consistent with findings presented in Table 2 in the main text, which demonstrated that limestones react in broadly similar ways in terms of colour change as they reach certain temperature thresholds. As a result, the limestone used provides indicative results for the potential cause of heating at Montastruc. An infrared laser digital thermometer (temperature range: -50 - 750°C; accuracy: ±2°C) was used to make all temperature recordings in each experiment. A Nikon D3500 camera fitted with a 18-55mm DX VR lens was used to photograph all aspects of the experiments. A Nikon D5500 camera fitted with a AF-S VR MICRO-NIKOR 105mm F/2.8G IF-ED macro lens was used to supplement photography of diagnostic heating and burning features on the replica plaquettes. Images of the experimental plaquettes were subsequently processed with the DStretch© plugin of ImageJ© using the LAB colourspace matrix to visualise discolouration caused by heating, allowing for a comparison with plaquettes from Montastruc. Observations of limestone changes were informed by published experimental and observational data, summarised in Table 2 in the main text. Qualitative observations were also recorded at all stages of each experiment and where possible documented via photography.

Experiment A: Taphonomy

To determine whether taphonomic processes may have caused unintentional burning on plaquettes, a number of replicas were placed in the vicinity of a fire. To simulate a scatter of plaquettes in varying conditions of deposition or burial, replicas were: (1) left exposed on the surface uncovered by sediment (surface uncovered: SU); (2) left on the surface but covered with a thin layer (<1cm) of sediment (surface covered: SC); (3) buried in the ground but with the top surface exposed (buried uncovered: BU); (4) buried in the ground and the top surface covered by sediment (buried covered: BC). The replica plaquettes were placed in a grid, with a plaquette of each configuration positioned at three different distances from a central fire: (1) directly underneath; (2) 35cm away; (3) and 70cm away (S.I. Fig. 1; S.I. Fig. 2). Distances were measured from the centre point of the grid, meaning that when the volume of the fire is taken into consideration - which changed throughout the experiment as it was fuelled - the true distance from fire to any given replica plaquette was variable, but always smaller than these stated distances. This yielded a sample of 12 replica plaquettes in total, one for each burial condition and distance.

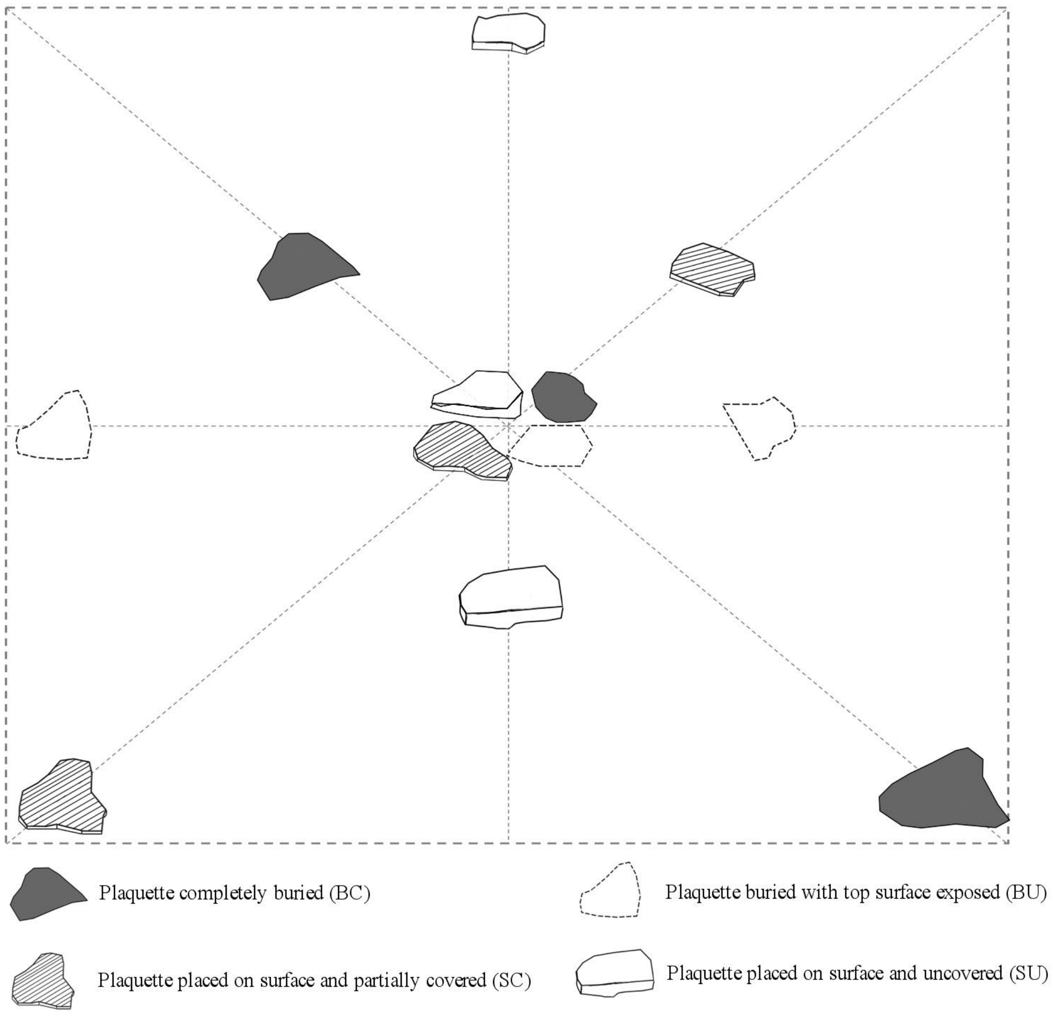

S.I. Fig. 1 Diagram showing the position of replica plaquettes and their burial configuration in relation to the position of the fire. The centre point of the image where the dashed lines converge marks the position of the fire. Total experiment area is 1.4m x 1.4m

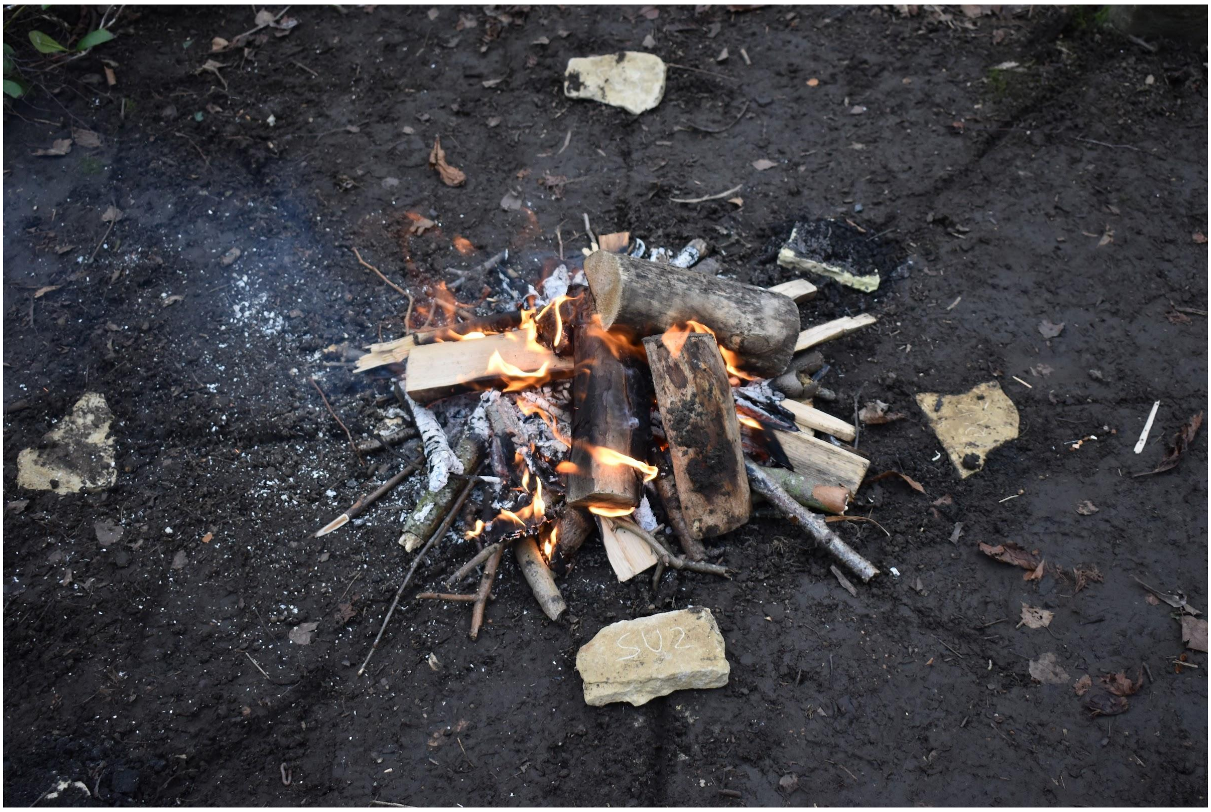

S.I. Fig. 2 Photograph showing the position of replica plaquettes in relation to the fire at the start of the experiment. Note that the most proximate plaquettes are not visible, having been entirely engulfed by the fire and fuel. Total experiment area is 1.4m x 1.4m

During the experiment the temperature of the fire and the ground at the 35cm and 70cm distances measured from the centre point of the grid were recorded at 10 minute intervals. The fire was fuelled as required to maintain it for 60 minutes, then left to die down. The plaquettes were left in place to cool for 24 hours. These protocols were then repeated, emulating heating and cooling cycles associated with the re-lighting of a fire in the same position. Plaquettes could not be disturbed during the experiment and many were obscured by sediment. As a result, temperature data was not recorded for plaquettes directly and photography was taken only after both heating cycles.

Experiment A: Taphonomy results

Experiment A results are summarised in S.I. Table 1, which describes the physical changes in the limestone in relation to their location and temperature, and S.I. Fig. 3, which shows the observed

physical changes in the limestone. The results indicate that the taphonomic scenario is unlikely to entirely explain the pattern of heating and burning on the Montastruc plaquettes. Only plaquettes that had a surface directly exposed to the central temperature of the fire reached high enough temperatures to cross the threshold required to cause observable colour and texture changes in the limestone.

S.I. Table 1

Direct contact with heat source			
Replica plaquette code	Distance from centre of the fire	Max. temperature of ground after 1 hour	Changes observed
SU1	0cm	702	Pink discolouration present across all surfaces. All surfaces also exhibited greyish discolouration and superficial lime formation.
SC1	0cm	702	Pink discolouration present across all surfaces, with patches of grey discolouration. Upper and lower surfaces exhibited bands of black soot.
BU1	0cm	702	Pink discolouration present across all surfaces, with patches of grey discolouration on the upper surface. Black soot is present across the majority of the lower surface.
BC1	0cm	702	Pink discolouration present across all surfaces.
Close to heat source			
Replica plaquette code	Distance from centre of the fire	Max. temperature of ground after 1 hour	Changes observed
SU2	35cm	27	No observable change to limestone
SC2	35cm	27	No observable change to limestone
BU2	35cm	27	No observable change to limestone

BC2	35cm	27	No observable change to limestone
**Distant from heat source**			
Replica plaquette code	Distance from centre of the fire	Max. temperature of ground after 1 hour	Changes observed
SU3	70cm	17	No observable change to limestone
SC3	70cm	17	No observable change to limestone
BU3	70cm	17	No observable change to limestone
BC3	70cm	17	No observable change to limestone

Table showing results of taphonomic heating experiments, noting the distance from the heat source and maximum temperature recorded at that position on the grid. Ground temperature is used as a proxy for intensity of heat to which plaquettes were exposed. Physical changes observed in the limestone for each replica plaquette are noted. All temperatures were measured in degrees celsius (°C)

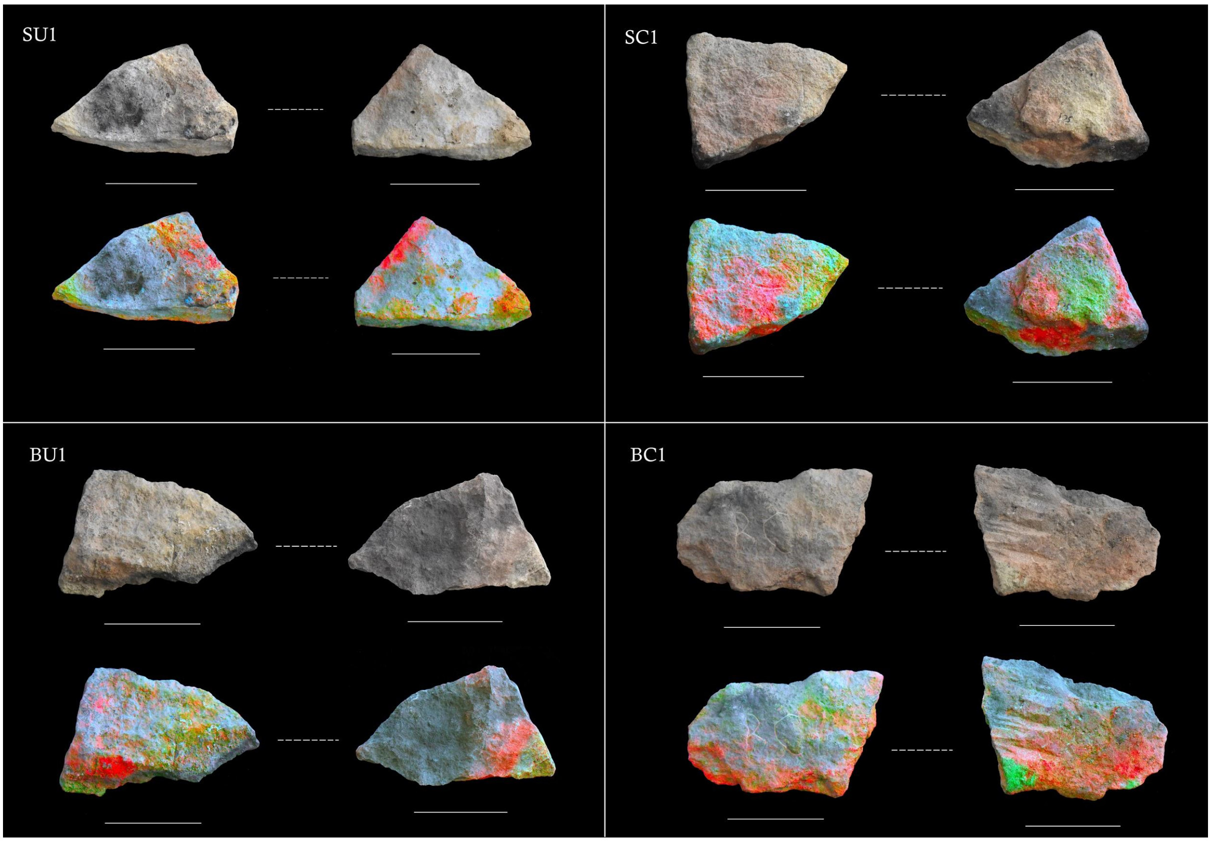

S.I. Fig. 3 Photographs showing patterns of heating and burning on the replica plaquettes in direct contact with the fire (SU1, SC1, BU1, BC1) via unmodified photographs (top of each panel) and the same photographs modified via DStretch© (bottom of each panel). Scale bar below each replica plaquette is 8cm in length

Where texture and colour changes did occur, this was dramatic and inconsistent with the heating signatures observed on the Montastruc plaquettes. For example, the plaquette (SU1) completely exposed to the fire partially changed to a grey colour with lime (CaO) formation on its upper surface. Lime begins to form on the surface of limestone at around 600°C and the internal structure deteriorates sharply between 700-800°C, causing total pulverisation of the stone [93, 94]. This type of discolouration and deterioration of the structure was not evident at Montastruc. Whilst pot lidding and cracking suggested some exposure to high temperatures, both were identified relatively infrequently on the Montastruc plaquettes.

Comparing plaquettes with exposed surfaces to those partially or completely buried demonstrated that a thin layer of sediment insulated the limestone against the heat of the fire. Plaquette SC1, placed on the surface of the ground with the upper surface partially covered by loose sediment (c. 0.5 - 1cm in depth) exhibited rubefaction across the upper surface but a grey colour on the exposed edges. Rubefaction occurs at around 100-300°C, changes to grey between 400 - 600°C and superficial lime formation occurs at the 600°C temperature threshold ([89]; see table 2, main text). While the sediment covering was of a minimal depth and uncompacted, this appeared to reduce the temperature to which the plaquette surface was exposed by a minimum of c. 100°C, based on the lack of superficial lime formation despite the temperature of the fire reaching above 700°C. Similarly, the plaquette (BU1) that was buried with the upper surface exposed, exhibited burning and a grey discolouration on the exposed surface, but only rubefaction and sooting on the buried surfaces. Soot is combusted at >400°C [125], further suggesting the buried surfaces were insulated against the extreme temperatures of the fire. The plaquette completely buried (BC1) c. 2-5cm average depth was consistent with these

findings, being further insulated from the heat with no evidence of superficial lime formation but exhibiting the presence of soot and rubefaction.

Plaquettes placed c. 35cm away from the centre of the fire did not exhibit any evidence of burning or heating. This is despite the nearest edges of the plaquettes placed at this distance being in proximity to the flames as the fire footprint spread. Plaquettes placed c. 70cm away from the centre of the fire also did not exhibit any evidence of burning, as these were too far away to directly come into contact with the hearth. This suggests that within the archaeological record, plaquettes would have had to be placed in very close contact with the fire to exhibit pronounced evidence of burning. Where heating and burning is evident on these plaquettes, it appears to be pronounced and homogeneous, particularly on exposed surfaces. This particular signature is inconsistent with the heating and burning pattern observed on the Montastruc plaquettes. However, as taphonomy is likely to be highly specific to any given site and collection of plaquettes, it must remain an important factor for any archaeological assemblage.

Experiment B: Boiling stones

To explore whether plaquettes may have functioned as boiling stones, in the manner of similarly identified stones in the Upper Palaeolithic and pre-Columbian North America [103-106], five plaquettes were placed in a fire and heated for 20 minutes; this was sufficient time for the plaquette temperatures to rise to between 150 - 400°C and plateau. Observed variations in temperature between plaquettes was linked to their size, with smaller plaquettes reaching higher temperatures than larger examples. On being removed from the fire, the temperature of the edge oriented towards the fire and the centre of the uppermost face of the limestone were recorded. The plaquette was then submerged in c. 5 litres of water contained within a metal bucket for safety (S.I. Fig. 4). The water temperature was measured both before and after each plaquette was submerged to assess the extent of temperature change for the first two cycles. After the plaquettes had sufficiently cooled within the water - defined

as when visible steam was no longer produced - they were removed and any visual changes to the limestone noted. The protocol was repeated three times for each plaquette, emulating repeated cycles of use.

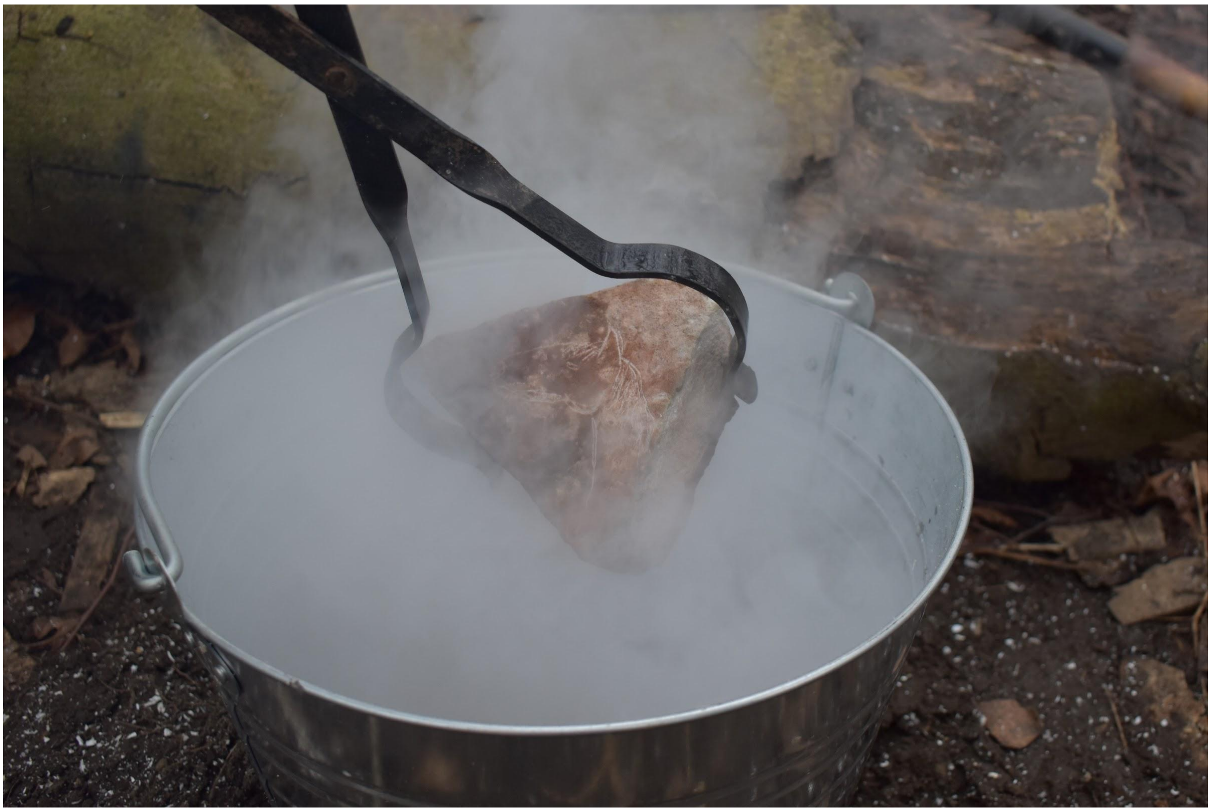

S.I. Fig. 4 Photograph showing replica plaquette PB4 after being submerged in water, in this case revealing a dramatic rubefaction

Experiment B: Boiling stones results

The results of Experiment B are presented in S.I. table 2, which summarises the temperature data collected across cycles, and S.I. Fig. 5, which summarises the observed physical changes in the limestone.

S.I. Table 2

Cycle 1						
Replica plaquette code	Plaquette edge temp. before	Plaquette edge temp. after	Plaquette central temp.	Plaquette central temp. after	Water temp. before	Water temp. after

			before			
PB1	335	89	44	23	16	18
PB2	398	56	95	49	21	20
PB3	356	80	63	39	20	20
PB4	330	69	86	51	20	20
PB5	213	58	98	44	20	21
**Cycle 2**						
Replica plaquette code	Plaquette edge temp. before	Plaquette edge temp. after	Plaquette central temp. before	Plaquette central temp. after	Water temp. before	Water temp. after
PB1	408	97	197	45	26	29
PB2	401	104	321	53	29	42
PB3	429	70	72	33	44	48
PB4	439	88	78	34	46	48
PB5	235	47	63	24	48	46
**Cycle 3**						
Replica plaquette code	Plaquette edge temp. before	Plaquette edge temp. after	Plaquette central temp. before	Plaquette central temp. after	Water temp. before	Water temp. after
PB1	376	34	122	49	n/a	n/a
PB2	402	85	240	110	n/a	n/a
PB3	520	32	365	3 6	n/a	n/a
PB4	460	88	503	80	n/a	n/a
PB5	303	25	380	61	n/a	n/a

Table showing temperature results over three cycles of heating and submerging replica plaquettes. Water temperature results are noted for the first two cycles to assess efficacy of the method. Temperatures measured in degrees celsius (°C)

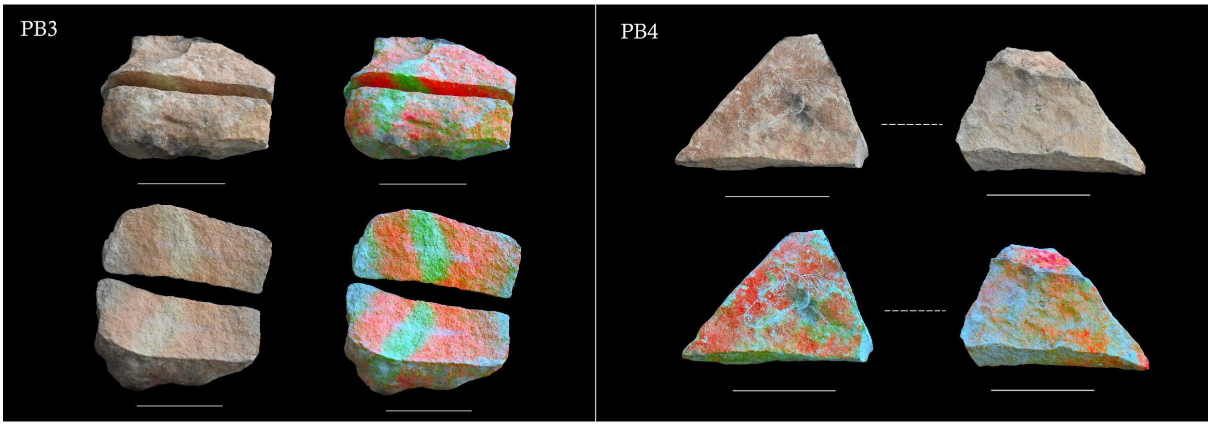

S.I. Fig. 5 Photographs showing heating and burning pattern on the boiling stones PB3 and PB4 after three cycles. The images on the left of panel PB3 show unmodified photographs, to the right the same photographs modified via DStretch©. The top of panel PB4 shows an unmodified photograph and the bottom of the panel the same photograph manipulated via DStretch©. Scale bar is 8cm in length

Significant changes in the colouration and integrity of the limestone were observed in the water boiling experiment. Plaquettes reached high temperatures, up to 400°C, before being rapidly cooled when submerged in water. All of the plaquettes used in this way exhibited pinkish - greyish discolouration, in keeping with expectations for limestone reaching or exceeding 100-300°C and 400-600°C temperature thresholds (see table 1, main text). As demonstrated in S.I. Fig. 5, the colour change was extensive and evident across all surfaces of the plaquette. These colour changes became particularly apparent after the plaquette was submerged in water. It is likely the water acted both to clean the surfaces of debris and soot, and saturate the stone to reveal the true extent of the vivid colour changes. The repeated process of heating and cooling weakened the integrity of the limestone via thermal shock, with several examples exhibiting cracking during the experiment. In one case, the plaquette (PB3) broke in two as a result of this process (S.I. Fig. 5). Another visual effect was the rapid driving off of water due to heat retained in the stone, which made the engravings appear momentarily distinct and prominently visible.

Raising water to boiling temperature proved ineffective, but water was heated and maintained to a temperature exceeding 40°C that could itself be useful for a range of activities [107, 108]. Heating time was slow, likely due to the volume of water used and the transferring of one stone at a time to allow for the recording of physical changes in the limestone. The temperature of the plaquette had an impact on the increase in water temperature. For example, the hottest plaquette (PB3), with a recorded temperature of 520°C, increased the water temperature by 6°C. Stones of a lower temperature prior to submersion had less impact on the temperature of the water.

The use of limestone plaquettes to heat water is technically possible and might have found application within a hunter-gatherer context. However, documented uses are rare except for two specific circumstances - the nixtamalization of maize, and the cooking of starchy foods in hot earth ovens [106, 166]. A previous experiment testing whether limestone fragments were possible boiling stones rejected this use after the stone disintegrated and formed a hydrated lime slurry in the experimental cooking water [104]. Alongside this, the shape of the plaquettes would be unusual if selected as boiling stones, as these are typically cobble shaped or rounded, even on the rare occasions where limestone might have been used [103, 105].

The action of heating and rapidly cooling plaquettes produced diagnostic heating, burning, and fracturing of the limestone that may be used to identify this activity in the archaeological record. The pattern generated does not appear to match the plaquettes from Montastruc specifically. While limestone may be unsuitable for heating water related to human consumption, this does not preclude heating using this method for other purposes. Heating and rapid cooling of limestone via submersion in water has demonstrable dramatic visual effects. It is possible these properties were recognised and integrated to provide an experiential aspect to the art, further explored in Experiment C.

Experiment C: Water and non-functional activities

In Experiment C, the effect of water on hot plaquettes was explored in terms of its dynamic visual effects, drawing inspiration from observations made during Experiment B and from other Palaeolithic art contexts where the addition of water was used to manipulate material properties; notably the exploding loess figurines reported from the Gravettian site of Dolni Věstonice (Czech Republic) [109-111]. The heating protocols used in Experiment B were repeated for Experiment C, with a sample of five plaquettes used. Plaquettes were placed in different positions within the hearth, typically with one edge in a closer proximity to the centre of the fire and another positioned towards the exterior to create a temperature gradient across the plaquette. Other plaquettes were positioned in yet closer proximity and orientated with more of their surface exposed to the centre of the heat source. Once the replica plaquettes reached a temperature plateau during heating, they were removed and water poured onto the engraved surface (S.I. Fig. 6) to explore whether: (1) plaquettes might have been used in this way to harness particular visual effects, and (2) if there were diagnostic differences in heating signatures between cycles of heating and full submersion in water (Experiment B) versus cycles of heating and controlled pouring of water onto the engraved surface only. The cycle of heating and water pouring was repeated three times to emulate extended use and to create comparative data with Experiment B. Any visual effects or physical changes were recorded via photography to allow for comparison with archaeological plaquettes.

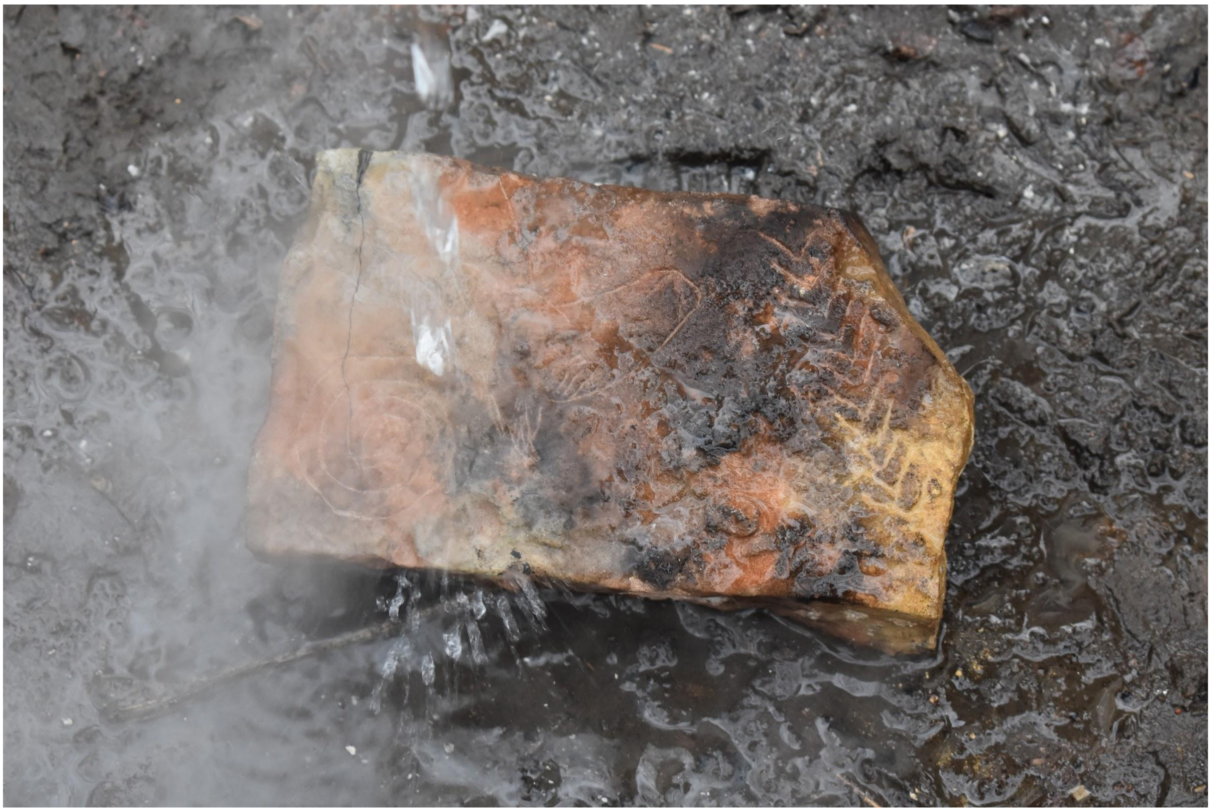

S.I. Fig. 6 Photograph showing replica plaquette WP3 being doused in water after heating. Note the vibrant colour change that occurs as the water saturates the limestone. Areas of engraving become momentarily more visible as the water rapidly evaporates due to the temperature of the rock.

Experiment C: Water and non-functional activities results

The results of Experiment C are presented in S.I. Table 3, which summarises the temperature data, and S.I. Fig. 7, which summarises the physical changes observed in the limestone.

S.I. Table 3

**Cycle 1**				
Replica plaquette code	Plaquette edge temp. before	Plaquette edge temp. after	Plaquette central temp. before	Plaquette central temp. after
WP1	308	141	60	56
WP2	182	73	89	77
WP3	150	63	45	43
WP4	337	119	444	254

WP5	278	186	153	94
**Cycle 2**				
Replica plaquette code	Plaquette edge temp. before	Plaquette edge temp. after	Plaquette central temp. before	Plaquette central temp. after
WP1	425	319	140	137
WP2	515	143	280	260
WP3	590	260	410	176
WP4	696	370	620	419
WP5	375	185	273	154
**Cycle 3**				
Replica plaquette code	Plaquette edge temp. before	Plaquette edge temp. after	Plaquette central temp. before	Plaquette central temp. after
WP1	357	88	231	105
WP2	235	94	187	96
WP3	173	88	226	105
WP4	357	190	393	93
WP5	160	94	406	260

Table showing results of heating replica limestone plaquettes over three cycles of heating and dousing with water. A significant and rapid reduction in temperature is evident with the addition of a small volume of water. Temperatures measured in degrees celsius (°C)

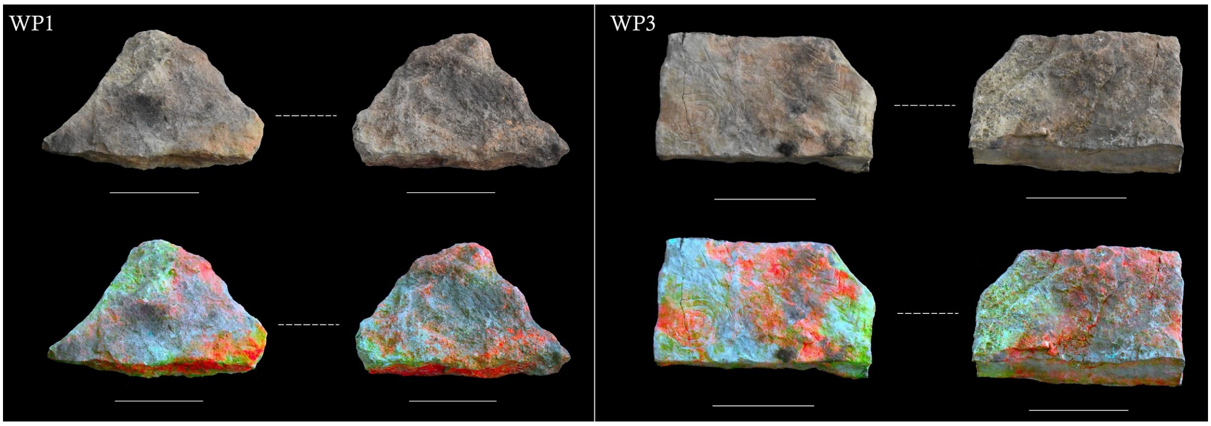

S.I. Fig. 7 Photographs showing the heating and burning pattern on replica plaquettes WP1 and WP3 after three cycles of heating and dousing with water. The top of each panel shows unmodified photographs, the bottom of each panel the same photograph manipulated via DStretch©. Scale bar below each replica plaquette is 8cm in length

In keeping with Experiment B, plaquettes reached temperatures ranging from between 150-450°C and colour change was particularly vivid on contact with water. Rubefaction was revealed in a dramatic fashion as the water was poured over the heated engraved surface, washing away debris and producing a 'sizzling' sound as water rapidly evaporated, causing a dramatic plume of steam. The engraved decoration momentarily appeared more vivid as the water evaporated from the plaquette surface.

The pattern of burning was varied but with a correspondence to the position of the plaquette within the hearth. In those cases where plaquettes were placed close to the edge of the hearth, the discolouration caused by heating was unevenly distributed across the surface. Edges exposed to the higher heat of the centre of the hearth exhibited a more pronounced colour change. After the first cycle, distinct bands of discolouration were observed on several plaquettes due to these differentials in temperature. Further, the addition of cold water to plaquette surfaces caused rapid cooling, reducing temperatures by up to 200°C. As a result of these rapid thermal fluctuations, particularly after several cycles of heating and cooling, plaquettes exhibited evidence of thermal fractures (see WP3, S.I. Fig. 7).

The heating signatures that resulted on the experimental examples shared features with the Montastruc plaquettes, with the bands of discolouration and thermal fracturing being comparable to those observed on the archaeological examples. Thermal fracturing appeared to result from the rapid cooling that occurred with pouring water onto the plaquettes, whilst bands of discolouration observed on some plaquettes were likely caused by the placement and orientation of the plaquettes in the hearth. Whilst there are similarities between the experimental and Montastruc examples, there is a notable disparity in the extent of the heating signatures. Notably, as the experimental plaquettes had to be partially or fully immersed within the hearth to heat the plaquettes to sufficient temperatures to

cause a dramatic effect when doused with water, the majority or all surfaces of the plaquette exhibited thermal modification. However, for the Montastruc plaquettes, discolouration and thermal modifications tend to favour one surface or edge. It is unlikely, therefore, that the Montastruc plaquettes were placed in the centre of a hearth to intentionally heat them to high temperatures, as with the experiment conducted here.

Experiment D: Oven structure

Informed by evidence from Magdalenian sites such as Étiolles [45] where plaquettes were used as part of the fabric of a hearth, and Monruz and Champréveyres (Switzerland) where stone was used to make structures associated with fires [99-101], Experiment E used replica plaquettes to construct an "oven" structure, stacking the limestone to create a semi-enclosed feature within which a fire was lit (S.I. Fig. 8).

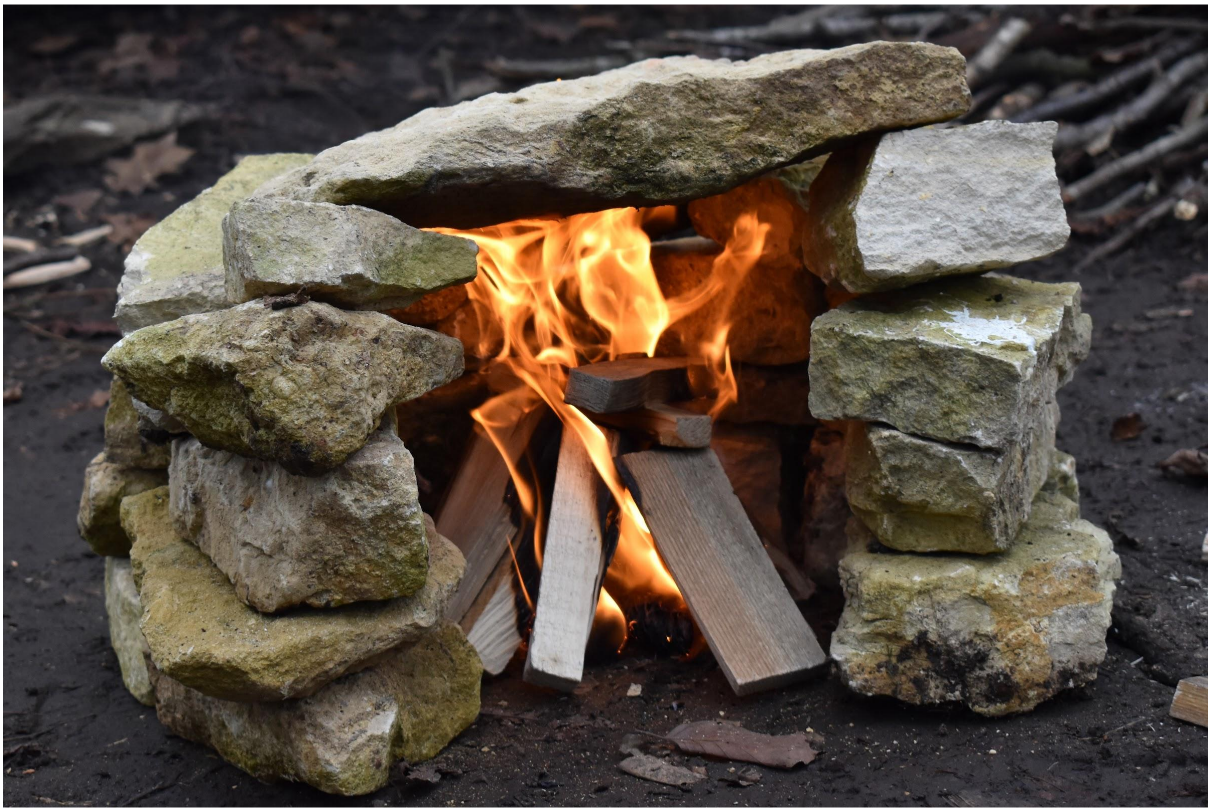

S.I. Fig. 8 Photograph showing the semi-enclosed oven structure used for experiment D

Amy’s [102] work demonstrated this use of plaquettes produced a functional and fuel efficient hearth, an important consideration during the Magdalenian where temperatures could be cold and wood fuel potentially scarce [100, 101]. The experiment carried out as part of this research therefore aimed to replicate Amy’s [102] experimental work and test this theory further as a possible practical function of plaquettes at Montastruc. A fire was lit inside the structure and temperatures were recorded for a sample of the stones facing the fire (the ‘inside’ surface) and the edge facing away from the fire (the ‘outside’ surface) for the top layer of stones (coded ‘A’) and the bottom layer of stones (coded ‘D’) from left (1) to right (6) (S.I. Fig 8). As the fuel obscured the view to some limestone surfaces inside the structure, a sample of visible surfaces across layers was used throughout the experiments. Refuelling sometimes obscured these data points and a measurement could not be taken. The temperature was recorded at ten minute intervals to explore how effective plaquettes were at retaining heat whilst the fire was active and being regularly fuelled (up to 60 minutes), and after fuelling ceased and the fire died down (70-80 minutes) and went out (at 90 minutes). The structure was left to cool for 24 hours before being deconstructed and visual changes on each plaquette were recorded. The plaquettes used in the experiment created a reference collection that could be compared against archaeological plaquettes to assess the extent of similarity and difference.

Experiment D: Oven structure results

The results of Experiment D are presented in S.I. Table 4, which summarises the temperature data, and S.I. Fig. 9, which details the physical changes observed in the limestone.

S.I. Table 4

Edge facings inside the structure							
Time (mins)	Capstone front edge	Stone inside 1	Stone inside 2	Stone inside 3	Stone inside 4	Stone inside 5	Stone inside 6
10	197	241	229	428	362	x	157
20	283	422	304	533	503	518	297

30	314	478	404	x	x	x	104
40	432	507	538	438	x	x	x
50	476	555	519	463	420	x	168
60	593	514	621	477	x	x	x
70	237	235	265	249	438	233	322
80	56	55	47	38	37	33	48
90	29	35	30	25	23	24	30
**Edge facings outside and top of the structure**							
Time (mins)	Capstone top	Stone outside A1	Stone outside A2	Stone outside A3	Stone outside A4	Stone outside A5	Stone outside A6
10	57	44	95	211	82	74	60
20	100	88	140	235	113	98	110
30	120	134	119	211	207	88	108
40	128	131	138	216	134	141	137
50	152	111	182	180	108	124	174
60	161	167	224	189	181	176	190
70	169	110	118	99	93	102	121
80	60	46	32	21	22	38	31
90	25	22	19	16	16	21	22
**Edge facings outside and bottom of the structure**							
Time (mins)	Capstone back edge	Stone outside D1	Stone outside D2	Stone outside D3	Stone outside D4	Stone outside D5	Stone outside D6
10	85	22	24	29	20	14	14
20	116	28	18	28	39	14	14
30	128	30	28	24	36	21	16
40	133	34	25	25	33	14	17
50	149	41	36	26	18	16	18
60	155	48	33	36	26	15	20
70	146	48	30	44	28	18	34
80	40	36	18	24	17	17	27
90	19	28	15	18	16	15	20

Table showing the temperatures of the oven hearth structure at differing positions, recorded at 10 minute time intervals. Plaquette codes refer to the position of the plaquette in the structure with the letter referring to height (A = uppermost layer to D = lowermost layer) and number referring to the position from left (1) to right (6). 'Capstone' is the large stone used to

cap the structure. 'Front edge' is the area proximate to the opening, 'back edge' the furthest edge from the opening. See S.I. Fig. 8 for relative positions. Temperatures measured in degrees celsius (°C)

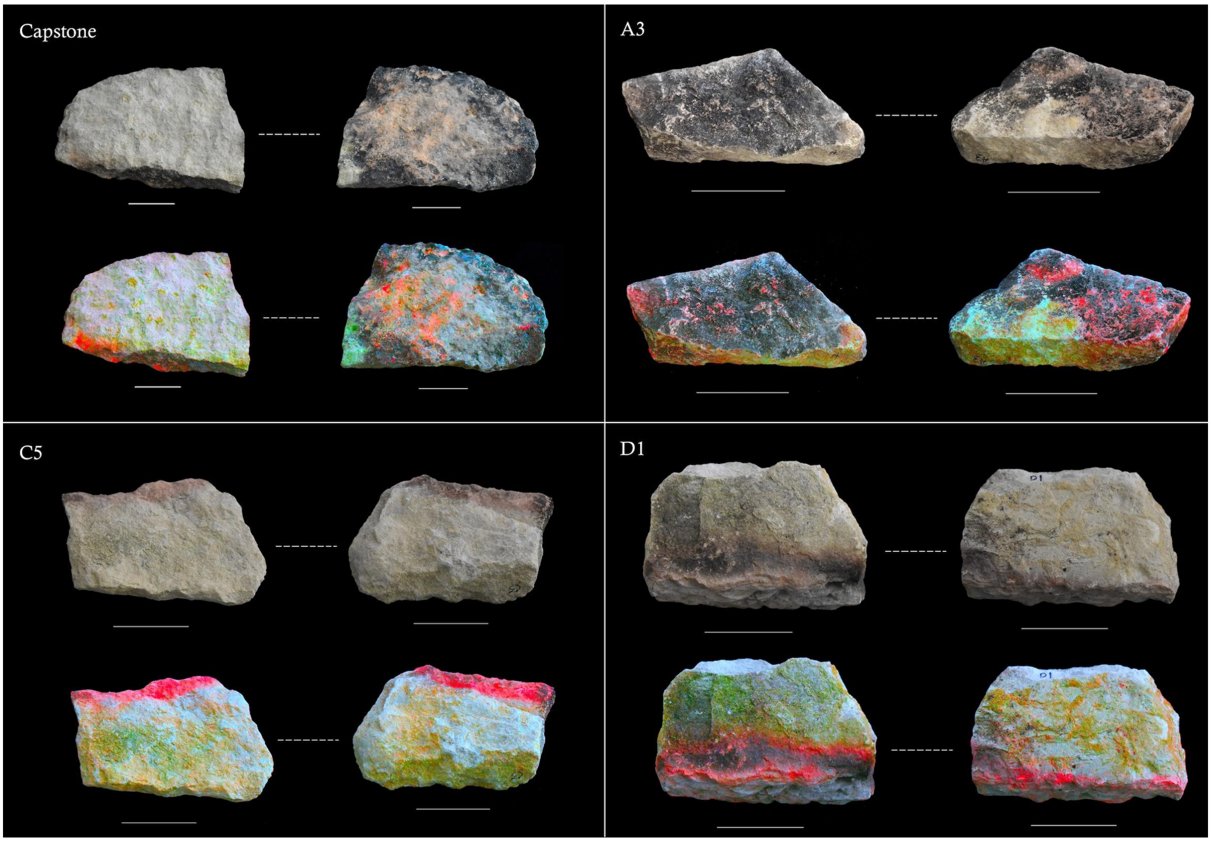

S.I. Fig. 9 Photographs showing heating and burning patterns on replica plaquettes used in the construction of the oven structure. Plaquette codes refer to the position of the plaquette in the structure with the letter referring to height (A = uppermost layer to D = lowermost layer) and number referring to the position from left (1) to right (6). The top of each panel shows unmodified photographs, the bottom of each panel the same photographs manipulated via DStretch©. Scale bar below each replica plaquette is 8cm in length

The construction of an oven structure using decorated plaquettes supports previous results (Amy 2020) demonstrating the effectiveness of stacked limestone blocks in creating a fuel-efficient hearth, with this configuration using less fuel than the open fires of the other experiments. Temperature of plaquette surfaces proximate to the heat source rose rapidly and after 60 minutes, a maximum temperature of 621°C was recorded. The internal edges of other stones facing the heat source could be several hundred degrees cooler than the highest recorded temperature at any given time as the fire

moved and changed within the structure. The temperatures of the stone edges furthest away from the heat source were consistently cooler, but did increase, with 235°C being the highest recorded temperature from the top layer of stones (A). The bottom layer of stones (D) in contact with the ground were, based on outside edge temperature measurements, cooler than those higher up the structure, with a significant variation from stone to stone depending on the movement of the flames. After the fire died down, the limestone held heat for several hours before returning to near ambient temperature. The flat capstone rapidly increased in temperature and was consistently hot, typically a little over 100°C, which would have been suitable for a range of tasks.

As the plaquettes remained fixed in place, only the internal edges and sides of the plaquettes were exposed to direct heat, resulting in a distinct pattern of heating and burning on the plaquettes. Discolouration on these internal edges was pronounced, with plaquettes exhibiting rubefaction and pronounced soot build up concentrated around small gaps between the layers of limestone (S.I. Fig. 10). Sooting can provide an indicator of temperature, being burned off as temperature increases (>400°C) [125]. It is likely the surfaces in direct contact with the fire had soot burned away (>400°C) and voids where smoke could escape became a trap for soot deposition. These areas show rubefaction, and when taken with the presence of soot, suggest a temperature of below 400°C. The heat radiated throughout the plaquette, but did not sufficiently heat the outer edges to meet the temperature threshold for a colour change to occur, resulting in a distinct gradation of discolouration across the plaquette. Surfaces not directly exposed to the fire showed no visually recognisable evidence of heating or burning, despite some external surfaces and reaching higher temperatures (<234°C). The result suggests external edges did not meet the c. 250°C temperature threshold for significant discolouration to occur, likely due to heat loss via radiation and the shifting heat source within the structure resulting in fluctuations and localised heating and cooling.

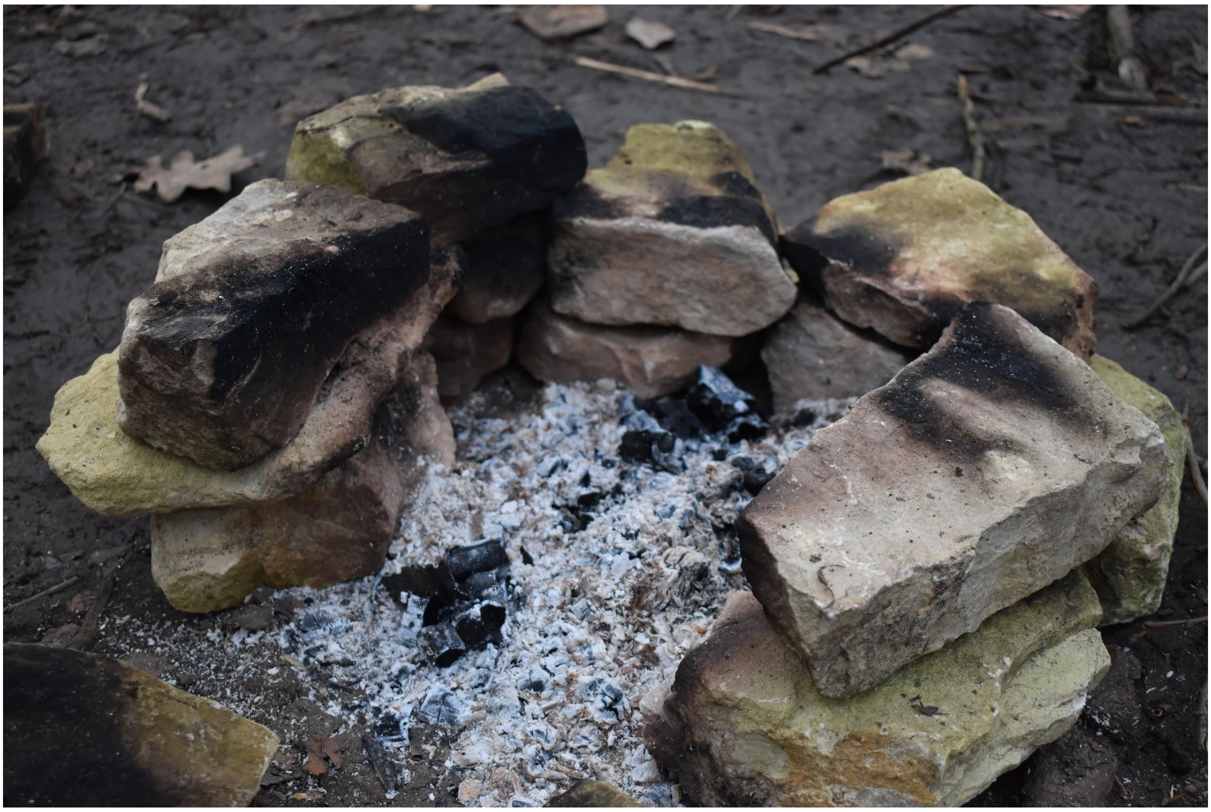

S.I. Fig. 10 Photograph showing partially deconstructed 'oven' hearth structure after the experiment, exhibiting the pronounced soot and discolouration on some plaquette surfaces. The internal edges were sufficiently hot (>400°C) for soot to burn off. Soot was deposited on surfaces below this temperature threshold and closely associated with block morphology, the spaces between blocks acting to channel airflow, in turn encouraging soot deposition. Outer edges furthest from the heat source show no modification

The bands of discolouration that resulted from this experiment, especially evident to the bottom layer of stones (D), were comparable to patterns of heating observed for the Montastruc plaquettes, with specific edges and surfaces appearing to be heated to higher temperatures than external edges. The more intensely modified pieces from the top of the structure are less similar to examples from Montastruc. The limestone used in constructing the oven structure required a flat shape for stability. For Montastruc, a number of the plaquettes might be unsuitable for this purpose, with several being too small or irregular in morphology. The intense sooting observed on some experimental pieces was not observed from examples derived from Montastruc, though it is possible adhering traces of this kind might not be preserved. It is interesting to note that the stacking of plaquettes in making the

structure served to entirely obscure the engraved surfaces. This does not preclude engraved plaquettes from such a use; the plaquette recovered from Ètiolles would have been similarly obscured [45]. However, it is unique amongst all experiments in making the art invisible during use in this way, with complex connotations for how the art might be understood, or perhaps suggesting a separation between use of the art and subsequent use in heating. Taken together, it is perhaps unlikely, therefore, that this configuration is responsible for the pattern of heating and burning on the Montastruc plaquettes.

Experiment E: Fireside at night

Parietal and portable art made in limestone caves was often created by lamp light in near darkness, causing particular visual and perceptual effects as the light cast by the lamp flickered across the decorated and morphologically complex cave surfaces [114, 115, 143, 164]. Experiment E was designed to provide a comparison to this experience of art in limestone caves by positioning plaquettes in close proximity to a fire at night, where the light of the fire similarly flickered over the surface of the engraved plaquette. Experiment E carefully positioned a sample of engraved and/or painted plaquettes at the edge of a hearth (S.I. Fig. 11) shortly after dusk, with low ambient lighting conditions. Experiment E was carried out in two phases: a preliminary phase where the experiment was run without collecting temperature data and focusing on visual observation and recording (FS1), and a second phase where the experiment was re-run to focus on collecting temperature data and to test and confirm visual observations (FS2). In both experiments plaquettes were positioned directly around the edge of the fire to form a ring. The fire was lit and fuelled for 60 minutes and observations recorded and documented via photography. The plaquettes were left in place for 24 hours, allowing for the plaquettes to be subjected to a cycle of heating and cooling.

Experiment E: Fireside at night results

The results of Experiment E are presented in Table S.I. 5, which shows temperature data from FS2 for plaquettes positioned in proximity to a hearth; S.I. Fig. 11, which shows example visual effects encountered during both rounds of the experiment; S.I. Fig. 12, which details the physical changes observed in the limestone from FS1; and S.I. Fig. 13, which details the physical changes observed in the limestone from FS2. Also pertinent are Supplementary Information files A-F, which show the VR simulations of several of the Montastruc plaquettes modelled around these experimental conditions.

S.I. Table 5

**FS2.1**			
Time (minutes)	Proximal edge	Central face	Distal edge
10	37.7	21.4	12.2
20	70.1	36.3	22.1
30	70.8	40.2	18.8
40	186.7	76.2	42.5
50	544.3	130.8	61.3
60	297.9	134.9	59.6
70	273.3	112.8	52.9
80	171.9	95.9	31.5
90	158	90.4	41.5
**FS2.2**			
Time (minutes)	Proximal edge	Central face	Distal edge
10	38	21	13.4
20	48.3	35.4	18.6
30	87.3	45.9	21.7
40	131.4	68.9	30.2
50	229.9	83.7	27.3
60	148.5	72	35.2
70	275.7	90.9	28.1
80	135.9	65.7	20.1
90	106	60.8	16.7
**FS2.3**			
Time (minutes)	Proximal edge	Central face	Distal edge

10	42.7	20.7	14
20	40.5	23.5	14.9
30	46.8	25.6	16
40	66.8	33.9	22.1
50	43.5	25.4	19.7
60	63	33.5	24.3
70	98	35.5	24.9
80	70.6	28.4	18.2
90	49.6	22	14
**FS2.4**			
Time (minutes)	Proximal edge	Central face	Distal edge
10	28	22.5	12.3
20	34.6	25.9	15.3
30	41.6	31.6	17.2
40	93.4	54.3	35.7
50	126	69.8	35.8
60	182.2	91.9	38.7
70	197.5	123.9	75
80	92	58.8	22.4
90	55.8	33.9	13.6
**FS2.5**			
Time (minutes)	Proximal edge	Central face	Distal edge
10	44.9	25.5	15.3
20	42.6	29.3	15.6
30	59.3	37.6	17.7
40	97.8	43.4	20.3
50	108.6	51.4	21
60	167.6	68.1	32
70	158.7	61.9	27.5
80	127.1	57.3	18.5
90	84.8	50.4	14.4
**FS2.6**			
Time (minutes)	Proximal edge	Central face	Distal edge
10	40.9	26.1	12.1

20	65.5	43.4	15.2
30	87.5	58.3	20.6
40	104.6	68.2	21.2
50	126.2	93.6	65.7
60	182.2	110.3	31.6
70	242.9	112.7	44.5
80	173.7	117.3	28.3
90	123.1	97.8	22.8

Table showing results of heating replica limestone plaquettes positioned in direct proximity to the edge of an open fire. Temperatures measured in degrees celsius (°C)

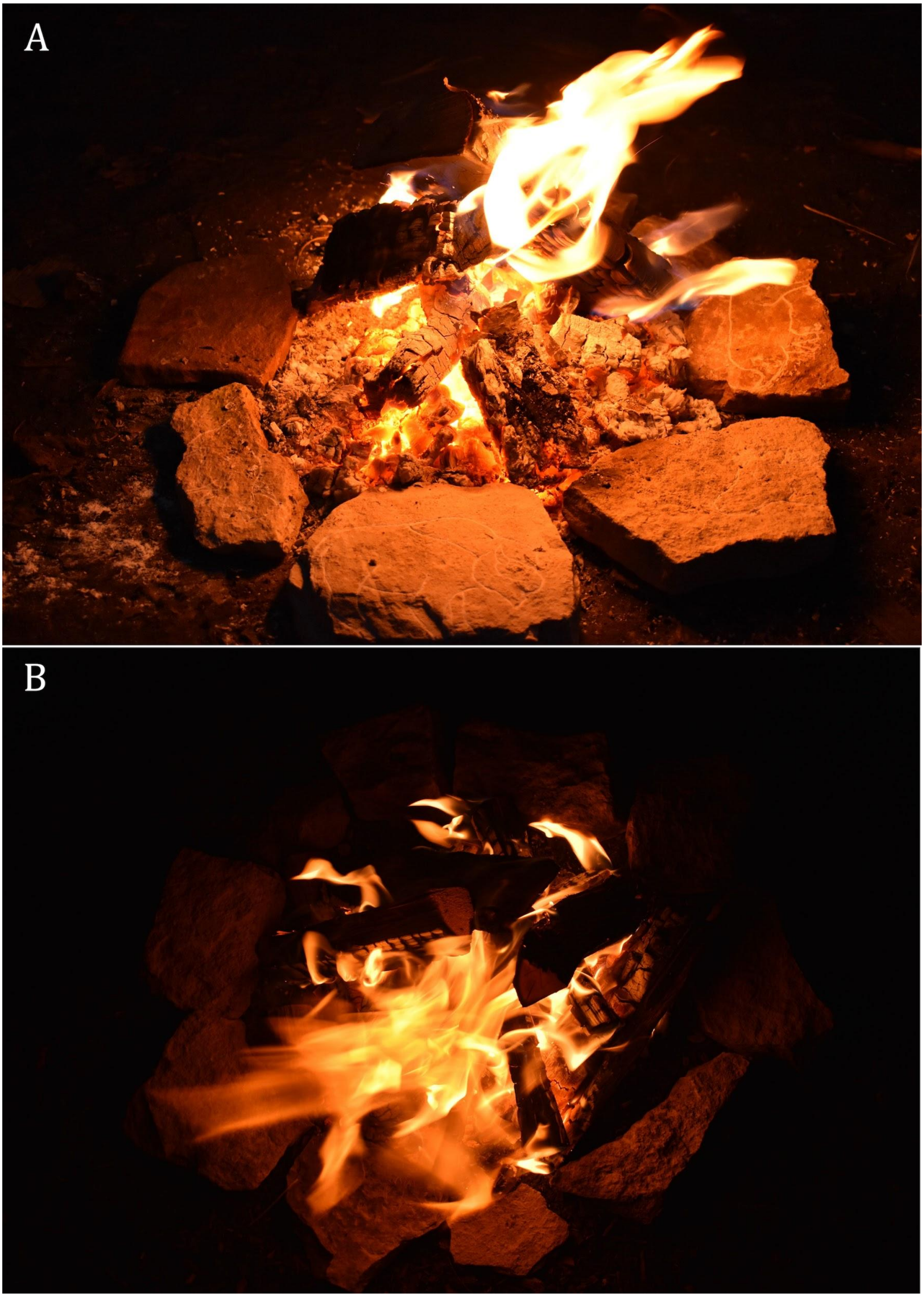

S.I. Fig. 11 Photograph showing decorated replica plaquettes placed by around the edge of a small hearth during A) FS1 and B) FS2, demonstrating some of the prominent visual effects of this configuration

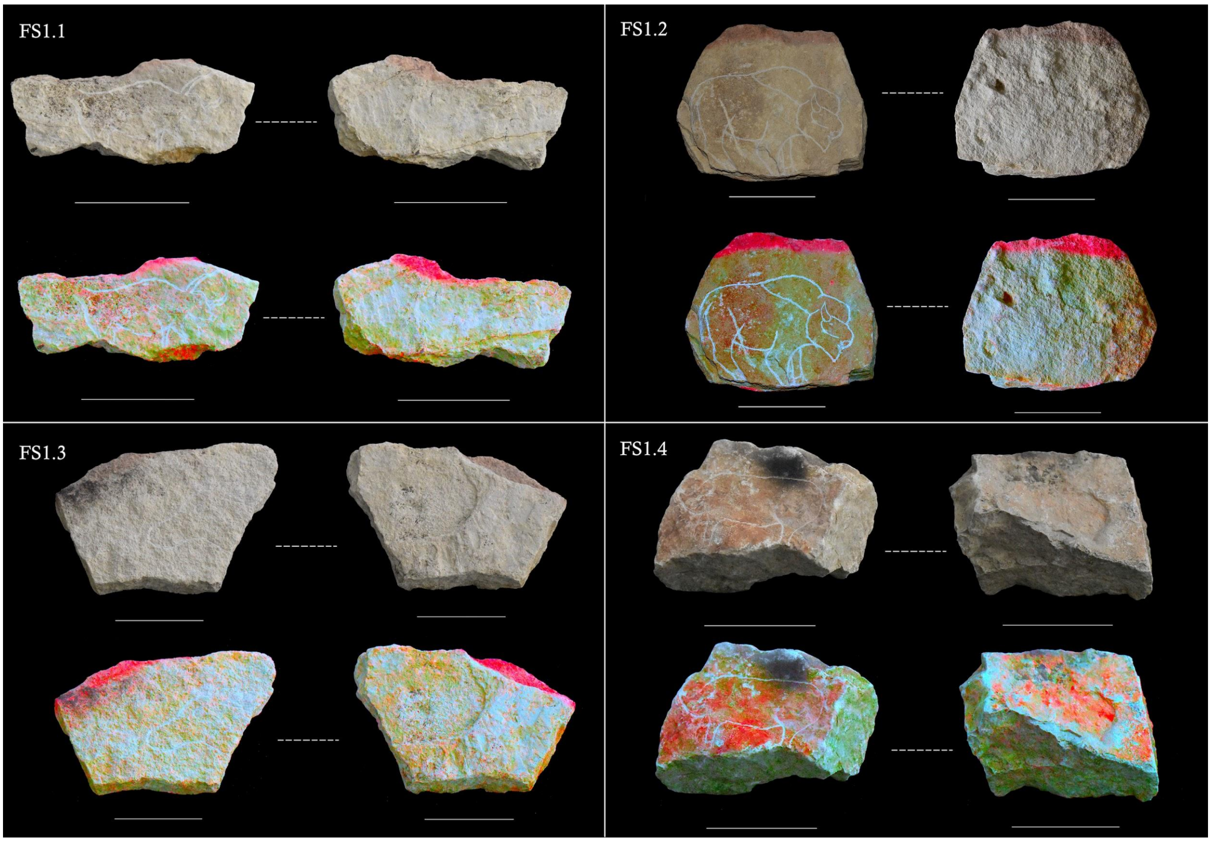

S.I. Fig. 12 Photographs showing heating and burning patterns on replica plaquettes FS1.1 - FS1.4. Note the variation in heating pattern caused by different placements around the hearth, with FS1.1 exhibiting minimal discolouration and FS1.4, which was partially engulfed by flames during the experiment, exhibiting more pronounced pink discolouration (see S.I. Fig. 11) and sooting, consistent with a temperature <400°C. The top of each panel shows unmodified photographs, the bottom of each panel the same photograph manipulated via DStretch©. Scale bar below each replica plaquette is 8cm in length

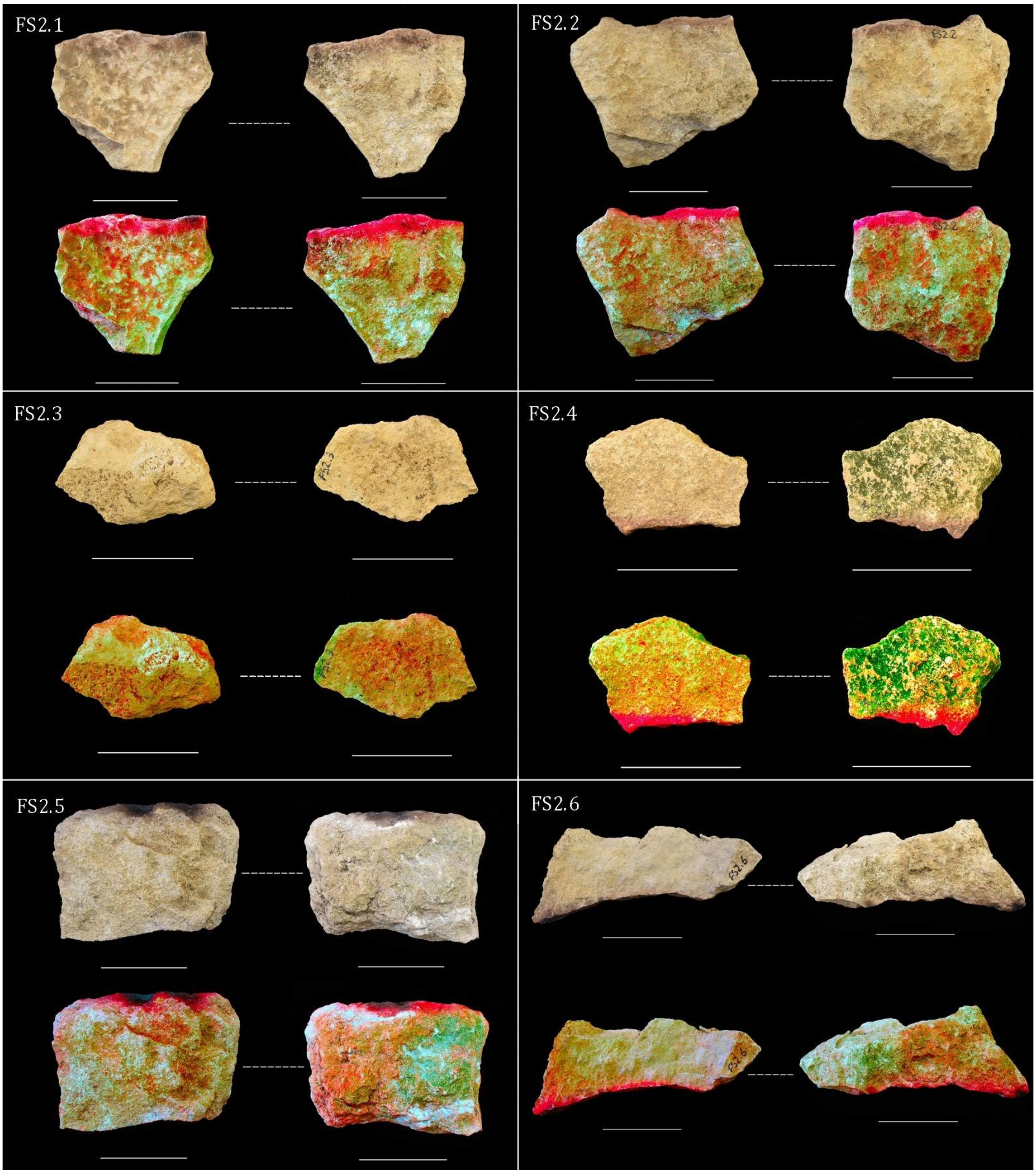

S.I. Fig. 13 Photographs showing heating and burning patterns on replica plaquettes FS2.1 - FS2.6. Note the variation in heating pattern, consistent with the temperature data and observations made in FS1.The top of each panel shows unmodified photographs, the bottom of each panel the same photograph manipulated via DStretch©. Scale bar below each replica plaquette is 8cm in length

As the plaquettes remained in place around the hearth, only one or two edges were exposed to the extreme heat of the fire, causing a pattern of burning and heating demarcated by rubefaction in these

localised areas of the plaquettes alone. Environmental factors were revealed to be an important consideration in the burning pattern produced on the plaquettes during these experiments. Each experiment was carried out in open air conditions and the wind gave an overall directionality to the heat created by the fire. Smaller scale changes to airflow and shifts in the positions of greatest heat as the experiments progressed was also observed. As reflected in the temperature data in S.I. Table 5, those plaquettes that were downwind of the heat source during FS2 showed evidence of burning around the edge, while those upwind of the heat source showed little appreciable change. These findings reinforce observations during FS1 where the same pattern was observed. In some cases, a dramatic difference in the order of hundreds of degrees celsius in temperature reached was observed (e.g. compare FS2.2, FS2.3, and FS2.4) between plaquettes in close proximity, depending on changes in wind direction and changes in fire intensity and morphology with refuelling. During FS1, FS1.4 was the plaquette subjected to the greatest intensity of heat. Rubefaction is evident across much of the surface and soot deposition is evident. These traces suggest the temperature of the plaquette did not exceed c.400°C and it is likely the temperature of the other plaquettes was lower than this, with the greatest intensity of heat localised to the immediate edges contacting the fire. During FS2, FS2.3 showed some of the greatest reactivity to changing light conditions (see Supplementary Information file G), but received the least exposure to heat that would leave a diagnostic archaeologically observable trace. FS2.1 was exposed to the highest heat, in this case above 500°C, burning away soot deposits and leaving a grey discolouration to the edge in proximity to the heat. Across FS1 and FS2, this produced a collection of plaquettes that superficially appear as though several were unmodified by heat, despite all of the plaquettes being distributed in close proximity to each other and to the fire. The heating and burning pattern across the replica plaquettes from these experiments closely resemble the dominant pattern of heating and burning observed on the Montastruc plaquettes.

Notable visual effects were observed during FS1 and FS2. The flames from the hearth cast light at an oblique angle across the plaquettes, causing elongated shadows that emphasised the natural contours

and features of the limestone and increased the visibility of the engraved decoration. However, this effect could be lost where the light was particularly strong after refuelling or where the plaquette was particularly flat and featureless. Equally, where engraving was shallow, the light could wash out and ambiguate a surface, while deeper engraving tended to give a more dramatic effect. This effect brought into focus the relationship between the engraved decoration and the morphology of the support. In some cases, it was clear where the natural features of the support had shaped the form of the engraved motif, for example with legs of an animal depiction being engraved onto natural contours of the support (see FS1.2, S.I. Fig. 11). The flickering nature of the light cast by the flames also caused additional visual and perceptual effects. The changing direction of the flames caused the light to continuously shift across the surface of the plaquettes, illuminating one plaquette and then another, but rarely illuminating all plaquettes at once. The dynamic movement of the light source gave the impression that the engraved animal depictions were moving, with features of the animals appearing to subtly shift position under the flickering light. This effect was explored for Montastruc via the use of VR, discussed in the main text, which showed similar visual effects perhaps indicating this was the method of use favoured at Montastruc (see Supplementary Information Files A-F).

Bibliography

166. Ellwood EC, Scott PM, Lipe WD, Matson RG, Jones JG. Stone-boiling maize with limestone: experimental results and implications for nutrition among SE Utah preceramic groups. Journal of Archaeological Science. 40(1);2013:35-44. https://doi.org/10.1016/j.jas.2012.05.044
